# Patient-related prognostic factors for function and pain after shoulder arthroplasty: a systematic review

**DOI:** 10.1186/s13643-024-02694-y

**Published:** 2024-11-22

**Authors:** Brechtje Hesseling, Nisa Prinsze, Faridi Jamaludin, Sander I. B. Perry, Denise Eygendaal, Nina M. C. Mathijssen, Barbara A. M. Snoeker

**Affiliations:** 1 Reinier Haga Orthopedic Center, Zoetermeer, The Netherlands; 2 Department of Orthopaedics and Sports Medicine, Erasmus MC, University Medical Center Rotterdam, Rotterdam, The Netherlands; 3 Department of Epidemiology and Data Science, University Medical Center Amsterdam, University of Amsterdam, Amsterdam, The Netherlands; 4 Medical Library Amsterdam UMC, Location AMC University of Amsterdam, Amsterdam, The Netherlands; 5 Department of Clinical Epidemiology and Orthopaedics, Lund University, Lund, Sweden

**Keywords:** Shoulder arthroplasty, Prognostic factor, Functional recovery, Pain, PROMs

## Abstract

**Background:**

While shared decision making is a cornerstone of orthopedic care, orthopedic surgeons face challenges in tailoring their advice and expectation management to individual shoulder arthroplasty patients due to the lack of systematically summarized evidence-based knowledge. This systematic review aims to provide an overview of current knowledge on independent predictive effects of patient-related factors on functional and pain-related outcomes after shoulder arthroplasty.

**Methods:**

We included longitudinal cohort studies including patients receiving total or reverse shoulder arthroplasty or hemiarthroplasty for primary osteoarthritis or cuff tear arthropathy. Studies with only univariable analyses were excluded. MEDLINE, Embase, and CINAHL databases were last searched on June 27, 2023. Risk of bias was evaluated using the QUIPS tool. For the analyses, we divided outcomes into three domains (Functional Recovery, Pain, and Functional Recovery & Pain) and four time points (short term, medium-short term, medium-long term and long term). When appropriate, meta-analyses were conducted to pool regression coefficients or odds ratios. Otherwise, results were summarized in a qualitative analysis. We used the GRADE approach to rate the certainty of the evidence.

**Results:**

Thirty-three studies analyzing over 6900 patients were included; these studied 16 PROMs and 52 prognostic factors. We could perform meta-analyses for six combinations of prognostic factor, domain, and time point. Only the meta-analysis for medium-long term poor ASES scores indicated worse outcomes for previous shoulder surgery (OR (95%CI) of 2.10 (1.33–3.33)). The majority of reported factors showed unclear or neutral independent effects on functional outcomes.

**Conclusions:**

Methodological heterogeneity and selective/incomplete reporting prevented us from pooling most results, culminating in a largely qualitative analysis. Depression, preoperative opioid use, preoperative ASES and SST scores, surgery on the dominant side, previous surgery, male gender, no. of patient-reported allergies, back pain, living alone, CTA vs OA, diabetes, and greater preoperative external ROM predicted neutral to worse or worse outcomes. In contrast, higher electrical pain threshold on the operative side, OA/RCA vs other diagnosis, and private insurance vs Medicaid/Medicare predicted neutral to better or better outcomes.

These results can help orthopedic surgeons tailor their advice and better manage expectations.

**Systematic review registration:**

PROSPERO CRD42021284822.

**Supplementary Information:**

The online version contains supplementary material available at 10.1186/s13643-024-02694-y.

## Introduction

Currently, it is a challenge for surgeons to provide evidence-based advice to their shoulder arthroplasty (SA) patients, since an overview of patient-related prognostic factors on functional and pain-related outcomes after shoulder arthroplasty does not exist. Thousands of patients undergo SA yearly, and the incidence continues to rise. For the United States, the incidence has been predicted to increase to at least 174.810 SAs for 2025 [1]. To each of these patients, orthopedic surgeons would ideally tailor their advice and expectation management regarding functional recovery and pain. After all, explaining evidence-based benefits and risks of treatment is an essential step in shared-decision making [2].

However, such an overview of the independent predictive effect of patient-related factors on outcomes after SA does not exist. Although Vajapey [3] described the influence of only psychological factors on outcomes after SA in a systematic review, the included studies mostly used univariable analysis or correlations and did not contain the full scope of relevant prognostic factors.

Since patient-related prognostic factors do not present in isolation as the outcome of surgery is determined by multiple factors, univariable analyses are insufficient to predict outcomes. Adjustment for other prognostic factors can affect not only the magnitude of the predictive effect but even the direction of the effect. To enable surgeons to tailor their advice to each individual patient’s circumstances, the first step is a thorough overview of evidence from studies investigating prognostic factors in multivariable analyses. To the best of our knowledge, no such overview is currently available in the literature.

Therefore, this systematic review aims to create an overview of the current body of evidence on the independent predictive effects of patient-related factors on functional and pain-related outcomes after SA.

## Materials and methods

### Eligibility criteria

We included longitudinal cohort studies with multivariable analysis to study patient-related prognostic factors on patient-reported pain and functional recovery after total shoulder arthroplasty (TSA), reverse shoulder arthroplasty (RSA), and hemiarthroplasty (HA). We only included studies with patients older than 18 years and in which at least 75% of included patients received SA for primary osteoarthritis or cuff tear arthropathy. If data could be extracted for SA alone, we also included studies comparing SA to another treatment. No restrictions were placed on the timing of outcome measurement.

Studies that were cross-sectional or case reports, only reported univariable analysis or correlations, included intra- or postoperative variables in the multivariable analysis, or used concomitant treatment were excluded.

### Prognostic factors under study

Any patient-related prognostic factor was of interest for this review, including but not limited to socio-demographic, physical, psychological, and work-related factors.

### Outcomes under study

For this review, only patient-reported outcome measures (PROMs) on pain and functional status were of interest. Measures with a component performed by a health professional (e.g., Constant-Murley score) were excluded. No limitation was placed on the timing of outcome measurement.

To aid in interpretation of the results, we grouped PROMS under three separate constructs:

- Functional recovery and pain—combined: American Shoulder and Elbow Surgeons score (ASES), Shoulder Pain and Disability Index (SPADI), Western Ontario Osteoarthritis of the Shoulder Index (WOOS), and Oxford Shoulder Score (OSS)
- Functional recovery—isolated: Quick Disabilities of the Arm, Shoulder and Hand score (QuickDASH), Disabilities of the Arm, Shoulder and Hand score (DASH), Simple Shoulder Test (SST), Patient-Reported Outcomes Measurement Information System (PROMIS) Upper Extremity (UE) subscale, PROMIS Physical Functioning (PF) subscale, PROMIS Pain Interference (PI) subscale, Single Assessment Numeric Evaluation (SANE), Shoulder Activity Scale (SAS), Subjective Shoulder Value (SSV), and ASES function score
- Pain—isolated: Visual Analogue Scale (VAS) pain and ASES pain score

Timing of the measurements are grouped under “short-term” (1 day to 3 months), “medium-short term” (3 months up to and including 1 year), “medium-long term” (after 1 year, up to and including 5 years) and “long term” (after 5 years).

### Search methods

The following databases were used up to July 5, 2021: MEDLINE (through PubMed), EMBASE (through Ovid), and CINAHL. A search update was performed on June 27, 2023, using the same strategy. A medical librarian (FJ) designed and performed a sensitive search strategy guided by the PICOTS format [4, 5] (Table 1). She deduplicated the results of both searches. For the full PubMed search strategy, see Table A1 in Appendix 1.

**Table 1:** PICOTS format

**Patients**	Patients who received HA, TSA, or RSA for primary OA or CTA
Index prognostic factors	Patient-related prognostic factors measured before the intervention
Comparator factor	-
Outcome	PROMs assessing functional recovery and pain
Time	Any range of prediction interval
Setting	Hospital care

*HA* Hemi-arthroplasty, *TSA* Total shoulder arthroplasty, *RSA* Reverse shoulder arthroplasty, *OA* Osteoarthritis, *CTA* Cuff tear arthropathy, *PROMs* Patient-reported outcome measures

### Data collection, data extraction, and risk of bias

All search results were imported into Rayyan, a web and mobile app for systematic reviews [6]. In Rayyan, two reviewers (NP, BH) independently screened titles and abstracts for eligibility and, subsequently, the full-text articles of studies that were deemed eligible. Next, for the included studies, the two reviewers independently extracted data on study characteristics, prognostic factors and outcomes and assessed the risk of bias (RoB) using the Quality in Prognostic Studies (QUIPS) tool [7]. When studies used more than one multivariable model to analyze their data, we extracted only the most complex model (i.e., adjusting for the highest number of factors).

For all stages, disagreements were solved by discussion and, if needed, a third reviewer (BS).

The QUIPS tool is divided into six domains: “study participation,” “study attrition,” “prognostic factor measurement,” “outcome measurement,” “adjustment for other prognostic variables,” and “statistical analysis and reporting.”

For the domain “adjustment for other prognostic variables,” we decided to only use moderate or high RoB, given the absence of a predefined evidence-based core set of variables to include in a multivariable analysis.

Studies received an overall “low RoB” rating when all six domains had low RoB, an overall “high RoB” rating when at least one domain had high RoB, and an overall “moderate RoB” rating when at least one domain had moderate RoB and none had high RoB.

For all stages, disagreements were solved by discussion and, if needed, a third reviewer (BS).

Extracted data and RoB results were recorded on a detailed Excel worksheet by each of the two reviewers, and a final version was created after discussion and reaching an agreement.

Since only studies that used multivariable analysis were eligible for this review, we extracted for each combination of prognostic factor and outcome either the β-coefficient or odds ratio (OR), its 95% confidence interval (CI), and its *p*-value. When data was unclear or missing, authors were contacted up to two times to request clarification by email.

If multiple reports for a single study were encountered, both reports were only used if they reported on different prognostic factor/outcome combinations.

### Quantitative data synthesis

For performing meta-analysis, a minimum of two studies per prognostic factor and outcome combination was necessary. First, we assessed whether clinical and methodological heterogeneity, including similar time points of outcome measurement, were sufficiently low to enable meta-analysis. If so, statistical heterogeneity needed to be acceptable (*I*^2^ < 75%) to pool results. We used random-effects models with Restricted Maximum Likelihood (REML) estimation [8, 9] to conduct the analyses, using R version 4.3.1 [10] in RStudio version 2023.6.1.524 [11] and the metafor package [12].

When multiple reports with the same source population were eligible for meta-analysis, we included the report with the lowest RoB or, in case of similar RoB, the largest sample size. We then performed sensitivity analyses by repeating the meta-analysis using the other study/studies instead, to examine whether this would substantively affect the results.

### Qualitative data synthesis

When meta-analysis was not possible, we performed a qualitative analysis. For the qualitative analysis, the following data from all studies were tabulated for each prognostic factor: the studied PROM, its method (e.g., raw score, change score) and timing of measurement, effect estimate with 95% CI and p-value, the number of other factors for which each study adjusted and overall RoB.

Because we did not find guidelines for defining or summarizing the direction of effect in a qualitative data synthesis of prognostic factor studies, we created and adhered to the following guidelines using the minimal clinically important difference (MCID) to determine the direction and size of effect:

- Better or worse outcomes: when all effect estimates and CI limits of involved studies exceeded either the MCID of that outcome (in case of an established MCID in the literature) or the neutral value (0 for linear regression coefficients and 1 for OR, when no established MCID was found) in a positive or negative direction, respectively
- Neutral to better/neutral to worse outcomes: when one end of the CI of at least one study exceeded the MCID or when no established MCID was found, the effect estimate of at least one study exceeded the neutral value after rounding
- Neutral direction of effect: when both CI limits of involved studies fell short of reaching the MCID or when no established MCID was found, the effect estimates of involved studies equalled the neutral value after rounding
- Conflicting direction of effect: when effect estimates or CIs of involved studies exceeded the MCID on both the positive and negative sides of effect
- Unclear direction of effect: when no effect estimates or only effect estimates without CI could be extracted from all involved studies (except when the effect estimate of at least one study exceeded the MCID, then “neutral to better/neutral to worse outcomes” was defined)

Table A2 in Appendix 1 lists the MCIDs we have utilized.

### Certainty of the evidence

We used the Grading of Recommendations, Assessment, Development and Evaluation (GRADE) approach to evaluate the certainty of the evidence [13], following the recommendations for the assessment of evidence about prognostic factors [14]. The certainty of the evidence can be rated down on the domains “risk of bias,” “inconsistency,” “indirectness,” “imprecision,” and “publication bias.” The certainty can be rated up when there is a clear dose–response gradient, a large effect, or when plausible confounding is present, adjustment for that confounding would increase the confidence in the found effect being the true effect. However, Foroutan et al. [14] report that they have not yet found examples of systematic reviews that mandate rating up for prognostic factors.

Since the interpretations of some aspects of GRADE are mainly described for quantitatively pooled estimates, we clarify our interpretation of the “imprecision” and “inconsistency” domains for use in our qualitative analysis as follows:

- Imprecision: Foroutan et al. [14] recommend evaluating imprecision by relating the upper and lower CI limits of the pooled estimate to a clinical decision threshold (in our case, the MCID). Because no pooled estimate is available in a qualitative analysis, we evaluated whether our conclusion on the overall direction of effect would change if the CIs of the individual studies were more precise (e.g., thereby changing from a CI with one end surpassing the MCID to a CI which falls short of reaching the MCID on both ends)
- Inconsistency: Foroutan et al. [14] state that to decide whether to rate down the quality based on inconsistency, “one should consider the contribution of the divergent study to the pooled estimate,” for example, by looking at the study’s weight in the meta-analysis. Instead, we evaluated the variability in point estimates and the extent of overlap in CIs of all involved studies

The GRADE approach was applied by two reviewers to each combination of prognostic factor and outcome construct when data from at least two studies could be extracted and resulted in one of the following levels of certainty (disagreements were solved by discussion):

- High certainty: further research is very unlikely to change our confidence in the association between the prognostic factor and the outcome construct
- Moderate certainty: further research is likely to impact our confidence in the association between the prognostic factor and the outcome construct
- Low certainty: further research is very likely to impact our confidence in the association between the prognostic factor and the outcome construct
- Very low certainty: any association between the prognostic factor and the outcome construct is very uncertain

Finally, for each construct, the overall judgments on effect and certainty of evidence for the prognostic factors were incorporated in Summary of Findings (SoF) figures, ordered per timing of measurements (medium-short term in the main text, other time points in the Appendix).

Since the GRADE approach is meant to rate certainty of aggregated evidence, prognostic factors for which the effect could only be based on a single study were incorporated separately into the SoF figures without a certainty rating. The same was done for prognostic factors for which the direction of effect was deemed unclear.

## Results

**Fig. 1:**
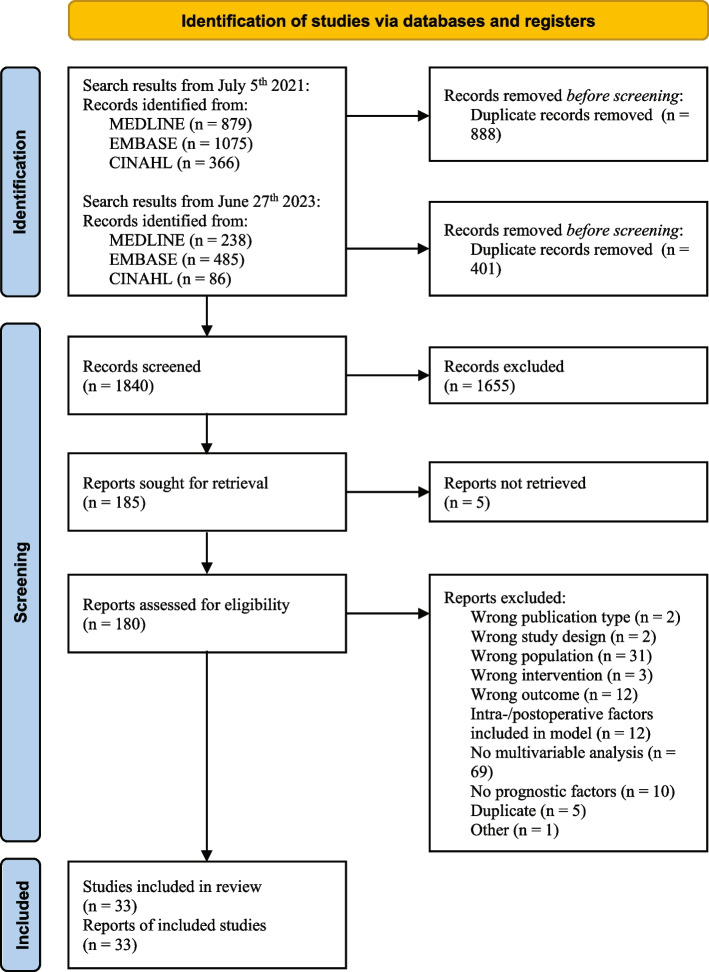
PRISMA flow diagram

After removing duplicates (*n* = 1289), the search resulted in 1840 articles. After screening titles and abstracts, 180 articles were retrieved for full-text screening. Five articles remained unavailable and were excluded [15–19]. In total, 33 studies were included in this systematic review [20–52]. For a complete overview, see the PRISMA flow diagram in Fig. 1.

### Included studies

Most of the included studies were (partly) conducted in the USA (*n* = 28). Of the 16 different PROMs studied in total, studies most frequently reported ASES (*n* = 25), VAS pain (*n* = 10), and SST (*n* = 7).

A total of 52 different prognostic factors were studied (in many different exact definitions), of which gender (*n* = 28), age (*n* = 25), and preoperative ASES score (*n* = 11) were the most frequently encountered. We did not find any results for short-term functional recovery and pain, short-term pain, or long-term pain.

Table 2 provides a detailed overview of all included studies.

**Table 2:** Characteristics of included studies

Study ID	Study design	Country, setting	Analyzed participants	Time point	Prognostic factors/factors adjusted for	Outcomes	Type of multivariable regression
Carducci 2019^a^ [20]	Prospective cohort	USA, New England Baptist Hospital, single surgeon	137 RSA patients with DJD, RCA or RCT, 2013–2016	Mean 29 months (± 8)	• RCA/RCT/DJD diagnosis • Preoperative ASES score • Opioid use • Previous shoulder surgery	• Poor ASES score • Bottom 30th percentile • Poor ASES improvement • Bottom 30th percentile	Logistic
Chang 2022 [21]	Retrospective cohort	USA + Canada, orthopedic departments, 25 surgeons in 23 centers	227 stemless TSA patients with GH arthritis (KL ≥ 3), recruitment period unknown	Min. 2 years	• Gender • Age • Preoperative ASES score	• Improved ASES score • No further definition	Logistic
Chawla 2022 [22]	Retrospective cohort	USA, University of Washington Medical Center, 3 surgeons	33 RSA patients and 39 CTA-H patients diagnosed with CTA, 2010–2013	Mean 2.2 years (± 0.3)	• Medicare/WC/Commercial insurance • Preoperative SST score (for CTA-H group only)	• SST raw score	Linear
Cho 2017 [23]	Prospective cohort	USA, Massachusetts General Hospital, single surgeon	46 TSA patients with GH arthritis, 2013–2015	1 year	• Gender • Age • HADS anxiety • HADS depression • Duration of symptoms • Involved side	• ASES raw score • SSV raw score • VAS pain raw score (0–10)	Linear
Dekker 2022 [24]	Retrospective cohort	USA, Steadman Philippon Research Institute, single surgeon	168 TSA patients with mostly primary OA, 2006–2016	Mean 4.6 years (2–12 years)	• Gender • Age • Glenoid retroversion • Glenoid inclination • Walch classification • Preoperative ASES score • Preoperative SANE score • Preoperative QuickDASH score • Subluxation	• ASES raw score • SANE raw score • QuickDASH raw score	Linear
Edwards 2020 [25]	Retrospective cohort	UK, Southmead Hospital, single surgeon	32 TSA patients with GH arthritis, 2006–2011	Mean 67 months (49–94 months)	• Fatty infiltration • Atrophy • Tendinopathy grade • Age • Gender	• OSS raw score • QuickDASH raw score	Linear
Fehringer 2002 [26]	Retrospective cohort	USA, University of Washington, single surgeon	102 TSA patients with GH arthritis, 1993–1997	30–60 months	• Preoperative SST score • Age • Gender	• SST raw score • SST change score • Change in no. of functions that could be performed expressed as percentage of no. that could not be performed before surgery • Change in ability to perform each of SST functions	General linear model analysis of variance
Forlizzi 2022^a^ [27]	Retrospective cohort	USA, New England Baptist Hospital, single surgeon	162 RSA patients with mostly OA or CTA, 2015–2018	Mean 28.3 months (± 8.1)	• OA/CTA/other diagnosis • Private/Medicaid/Medicare insurance • No. of patient-reported allergies • Depression • Previous surgery • Preoperative ASES score • Preoperative opioid use	• ASES top vs bottom quartile	Logistic
Franovic 2020 [28]	Retrospective cohort	USA, Henry Ford Health System, single surgeon	73 RSA patients with CTA, recruitment period unknown	Mean 9.6 months (± 5.0)	• Preoperative PROMIS-UE score • Preoperative PROMIS-PI score • Preoperative PROMIS-D score • Age • Gender • BMI • ASA	• MCID PROMIS-UE • MCID PROMIS-PI • MCID PROMIS-D	Logistic
Friedman 2018 [29]	Retrospective cohort	USA, University of Florida and NYU Langone Orthopedic Hospital, 13 surgeons	660 RSA patients with CTA or OA with RC tear, 2007–2014	Variable time points (range < 3–96 months)	• Gender • Age	• ASES change score • SST change score • SPADI change score	Linear mixed model
Green 2020 [30]	Retrospective cohort	USA, Brown University, single surgeon	176 TSA patients with advanced OA, recruitment period unknown	2 years	• Mean preoperative expectation score • Gender • Age • Dominant side • College education • Work status • Comorbidities • Depression/anxiety • Preoperative DASH score • Preoperative SST score • Preoperative VAS pain	• DASH raw score • DASH change score • SST raw score • SST change score • VAS pain score • VAS pain change	Linear
Huber 2020 [31]	Retrospective cohort	International, 5 clinics specialized in shoulder surgery (3 in Germany, 2 in France, 1 in Switzerland)	168 RSA patients with cuff arthropathy, recruitment period unknown	2 years	• Hamada grade • Age • Gender • Dominant side • ASA score	• ASES TE	Linear
Kadum 2018^b^ [32]	Prospective cohort	Sweden, Sundsvall Teaching Hospital, 2 surgeons	63 TSA patients with OA, 2014–2016	1 year	• Gender • Age • Preoperative electrical pain threshold (operative side and contralateral side) • Preoperative pain at rest • Preoperative pain on exertion • Preoperative QuickDASH score	• QuickDASH raw score	Linear
Kohan 2020 [33]	Retrospective cohort	USA, Washington University, 4 surgeons	111 TSA patients with OA, 2015–2017	Mean 20.5 months (12–29 months)	• Glenoid concentricity • Anxiety • Depression • Gender • Age • CCI • Smoking status • Dominant side	• ASES raw score • WOOS raw score • Pain on 0–10 scale • PROMIS-UE raw score • PROMIS-PF score • PROMIS-PI score	Linear
Lansdown 2021^e^ [34]	Prospective cohort	USA, University of California, 4 surgeons	491 TSA/RSA/HA patients with mostly OA/CTA, 2012–2017	Min. 2 years	• Age • BMI • Gender • CCI • Private/Medicare/Medicaid insurance • Zip code-based income	• ASES raw score	Linear
Lapner 2015 [35]	Retrospective cohort	Canada, multicenter	62 TSA patients with OA, 2006–2009	1 year	• Preoperative SSP FI • Preoperative SSP occupation area • Preoperative SSP atrophy grade • Preoperative ISP FI • Preoperative ISP atrophy grade • Age • Gender • Hand dominance • Preoperative SSP strength • RA vs OA	• ASES raw score • WOOS raw score	Linear
Matsen 2019 [36]	Retrospective cohort	International, multicenter (11 centers)	691 TSA patients with OA, 2000–2016	1 year and 2 years	• Gender • Preoperative glenoid version (continuous) • Preoperative glenoid anteversion/retroversion/ • neutral version • Previous surgery • Gender • Age • Walch classification • Preoperative ASES score • Preoperative SST100 score	• ASES raw score • ASES %MPI • SST100 raw score • SST100%MPI	Linear
McFarland 2021 [37]	Retrospective cohort	USA, Johns Hopkins University, single surgeon	44 RSA patients, 2009–2016	Mean 34 months (24–62 months (SSDI group)), mean 36 months (24–96 months (WC group))	• 2 or more previous surgeries • Age • Gender • Dominant side • College education • Marital status • Smoking • BMI • Comorbidities • Level of activity at work	• Poor ASES score (cut-point unknown)	Logistic
Moverman 2021^a^	Retrospective cohort	USA, New England Baptist Hospital, single surgeon	480 TSA/RSA patients with DJD/RCA, 2015–2018	2 years	• Presence of functional somatic disorder • No. of patient-reported allergies • Gender • Anxiety • Chronic preoperative opioid use • Diagnosis • Preoperative ASES score • Preoperative SANE score • Preoperative VAS score	• ASES raw score • SANE raw score • VAS pain raw score	Linear
Okoroha 2019^d^ [39]	Retrospective cohort	International, multicenter (3 in USA, 1 in France)	2364 TSA/RSA patients with mostly OA/RCT/RCA, 2007–2015	Mean 45.9 months (± 23.7 (women)), 46.4 months (± 23.6 (men))	• Gender • Age • BMI • History of surgery • Diagnosis	• ASES raw score • SST raw score	Linear
Patel 2019^d^ [40]	Retrospective cohort	International, multicenter (3 in USA, 1 in France)	1135 TSA patients with OA, 2006–2015	Mean 4.2 years (± 2.3 (age < 55)), 4.3 years (± 2.4 (age > 55))	• Age • Gender • BMI • Previous surgery • Diagnosis	• ASES raw score • ASES change score • SST raw score • SST change score • SPADI raw score • SPADI change score • VAS pain raw score • VAS pain change score	Linear
Pettit 2022^a^ [41]	Retrospective cohort	USA, New England Baptist Hospital, single surgeon	197 RSA patients with OA, 2015–2018	Mean 28 months (± 7.6)	• Age • Gender • Previous shoulder surgery • Preoperative ASES score • Walch classification	• ASES raw score	Linear
Polce 2021 [42]	Retrospective cohort	USA, Rush University, 5 surgeons	105 RSA patients with OA/CTA/mRCT, 2016–2018	Min. 2 years	• Gender • WC/other insurance • Diabetes mellitus • Dominant side	• ASES %MOI • SANE %MOI	Logistic
Rauck 2018^c^ [43]	Retrospective cohort	USA, Hospital for Special Surgery, multiple surgeons	137 RSA patients with mostly OA/CTA, 2008–2015	2 years	• Total no. of preoperative expectations • Expectation “Relieving nighttime pain” • Expectation “Improving nonoverhead sports” • *Age* • *Gender* • *SF-36 subscales* • *Preoperative VAS pain score* • Preoperative VAS fatigue score • Preoperative VAS general health score	• ASES raw score • SAS raw score • VAS pain raw score • VAS pain change score	Linear
Saini 2022^a^ [44]	Retrospective cohort	USA, New England Baptist Hospital, single surgeon	311 RSA patients with OA/CTA, 2015–2018	Mean 28.1 months (± 7.6 (OA)), 27.6 months (± 7.3 (CTA))	• Age • Gender • Previous surgery • Preoperative ASES score • Diagnosis	• ASES raw score	Linear
Sayed-Noor 2018^b^ [45]	Prospective cohort	Sweden, Sundsvall Teaching Hospital	63 TSA patients with OA, 2014–2016	3 months 1 year	• Gender • Age • Preoperative SSP FI • Preoperative SSP atrophy • Preoperative ISP FI • Preoperative ISP atrophy • Preoperative QuickDASH score	• QuickDASH raw score	Linear
Shields 2017 [46]	Retrospective cohort	USA, Beaumont Health, single surgeon	272 RSA patients with CTA/OARCT/mRCT, 2007–2014	Mean 25 months (± 13 (Previous RCR)) Mean 26 months (± 13 (Control))	• Previous RCR • Age • Gender • BMI	• ASES raw score • ASES change score • ASES ADL raw score • ASES ADL change score • SSV raw score • SSV change score • VAS pain raw score • VAS pain change score	Linear
Somerson 2016 [47]	Retrospective cohort	USA, University of Texas Health Science Center San Antonio, 2 surgeons	42 HA patients with CTA, 1991–2007	Mean 48 months (24–132 months)	• Preoperative active external rotation • Preoperative SST score • Preoperative VAS pain score • Tear limited to SSP and ISP	• 30% of MPI in SST score	Logistic
Strotman 2020 [48]	Retrospective cohort	USA, Loyola University Medical Center	91 TSA/RSA patients with mostly OA/CTA, 2013–2016	Min. 1 year	• Private/Medicare/WC/Medicaid insurance • Preoperative ASES score	• ASES raw score • VAS pain raw score	Linear mixed model
Swarup 2017^c^ [49]	Retrospective cohort	USA, Hospital for Special Surgery, multiple surgeons	67 TSA patients with OA, 2007–2008	2 years	• No. of “Very important expectations” • *Age* • *Gender* • *Preoperative VAS pain score* • *Preoperative VAS general health score* • *SF-36 subscales*	• ASES raw score • ASES change score • VAS pain raw score • VAS pain change score	Linear
Werner 2016^c^ [50]	Retrospective cohort	USA, Hospital for Special Surgery	490 TSA/RSA patients with GH arthritis/CTA, 2007–2013	2 years	• Preoperative ASES score • Living alone • Comorbidity back pain • Diagnosis	• MCID ASES score • SCB ASES score	Logistic
Werner 2017^c^ [51]	Retrospective cohort	USA, Hospital for Special Surgery	264 TSA patients with GH arthritis, 2007–2013	2 years	• Depression • *Age* • *Gender* • *ASA* • *Previous surgery* • *Subscapularis management* • *RC status* • *Total no. of comorbidities* • *BMI* • *Preoperative ASES score*	• ASES change score	Linear
Wong 2017^e^ [52]	Prospective cohort	USA, University of California, 3 surgeons	117 RSA patients with RCA/OARCT, 2009–2015	Min. 1 year	• Gender • BMI • Age • CCI • Dominant side • Smoking	• ASES function raw score • ASES pain raw score • ASES raw score • VAS pain raw score	Linear

For factors in italic print, it is unknown which factors were retained in the final model

*(m)RCT* (massive) rotator cuff tear, *(Quick)DASH* (Quick) Disabilities of Arm; Shoulder and Hand, *ASA* American Society of Anesthesiologists, *ASES* American Shoulder and Elbow Surgeons, *BMI* Body mass index, *CCI* Charlson Comorbidity Index, *CTA* Cuff tear arthropathy, *CTA-H* Cuff tear arthropathy—hemiarthroplasty, *DJD* Degenerative joint disease, *FI* Fatty infiltration, *GH* Glenohumeral, *HA* Hemiarthroplasty, *HADS* Hospital Anxiety and Depression Scale, *ISP* Infraspinatus, *KL* Kellgren & Lawrence, *MCID* Minimal clinically important difference, *MOI* Maximal outcome improvement, *MPI* Maximal possible improvement, *OA* Osteoarthritis, *OARCT* Osteoarthritis with rotator cuff tear, *OSS* Oxford Shoulder Score, *PROMIS-D* Patient-Reported Outcomes Measurement Information System—Depression, *PROMIS-PF* Patient-Reported Outcomes Measurement Information System—Physical Functioning, *PROMIS-PI* Patient-Reported Outcomes Measurement Information System—Pain Interference, *PROMIS-UE* Patient-Reported Outcomes Measurement Information System—Upper Extremity, *RA* Rheumatoid arthritis *RC* Rotator cuff, *RCA* Rotator cuff arthropathy, *RCR* Rotator cuff repair, *RSA* Reverse shoulder arthroplasty, *SANE* Single Assessment Numeric Evaluation, *SCB* Substantial clinical benefit, *SF-36* 36-Item Short Form Health Survey, *SPADI* Shoulder Pain and Disability Index *SSDI* Social security disability insuranc, *SSP* Supraspinatus, *SST* Simple Shoulder Test, *SSV* Subjective Shoulder Value, *TE* treatment effects, *TSA* Total shoulder arthroplasty, *USA* United States of America, *UK* United Kingdom, *VAS* Visual Analogue Scale, *WC* Workers’ compensation, *WOOS* Western Ontario Osteoarthritis of the Shoulder

^a^Forlizzi 2022, Moverman 2021, Pettit 2022, and Saini 2022 appear to have used the same source population (Carducci 2019 partly)

^b^Kadum 2018 and Sayed-Noor 2018 appear to have used the same source population

^c^Werner 2016 and Werner 2017 appear to have used the same source population and possibly overlap with Rauck 2018 and Swarup 2017

^d^Okoroha 2019 and Patel 2019 appear to have (partly) used the same source population

^e^Lansdown 2021 and Wong 2017 appear to have partly used the same source population

Several authors appeared to have used the same source population for their studies; these have been indicated in Table 2. These related study reports were not analyzed together in any meta-analysis.

Taking these related source populations into account, over 6900 patients were analyzed in the included studies.

### Risk of bias

**Fig. 2:**
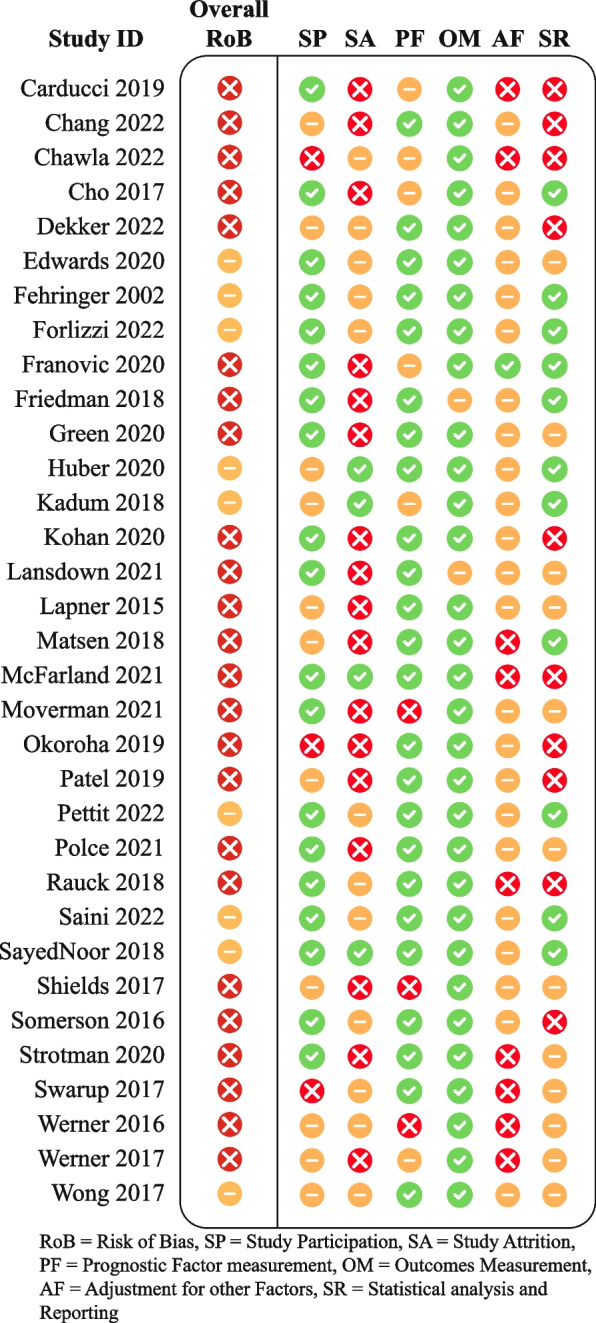
Risk of bias overview

Of the 33 studies, 9 scored moderate RoB, and 24 scored high RoB. No study scored low RoB. Moderate and high RoB were primarily due to the domains “study attrition” (e.g., excluding patients that were lost to follow-up (LtFU) where LtFU could be related to the prognostic factor or outcome), “adjustment for other factors” (e.g., unclear for which factors the final analysis adjusted), and “statistical analysis and reporting” (e.g., reporting only effect estimates without CI or *p*-value, only *p*-value, or not reporting any data at all). For the RoB overview, see Fig. 2.

### Meta-analyses

There was a high degree of methodological heterogeneity: outcomes were measured using different methods even when the same PROM was used. For example, male gender as prognostic factor for ASES scores was studied with raw scores, change scores, treatment effects, percentage of maximal possible improvement, and dichotomized scores. Therefore, we could only pool results for six combinations of prognostic factor and outcome measurements for the domain “Functional Recovery & Pain”: one for medium-short term (age on raw ASES scores [23, 36]) and five for medium-long term (male gender [36, 41], age [36, 44], Walch A2 vs A1 [36, 41], and previous surgery [36, 44] on raw ASES scores and previous surgery on poor ASES scores [27, 37]).

**Fig. 3:**
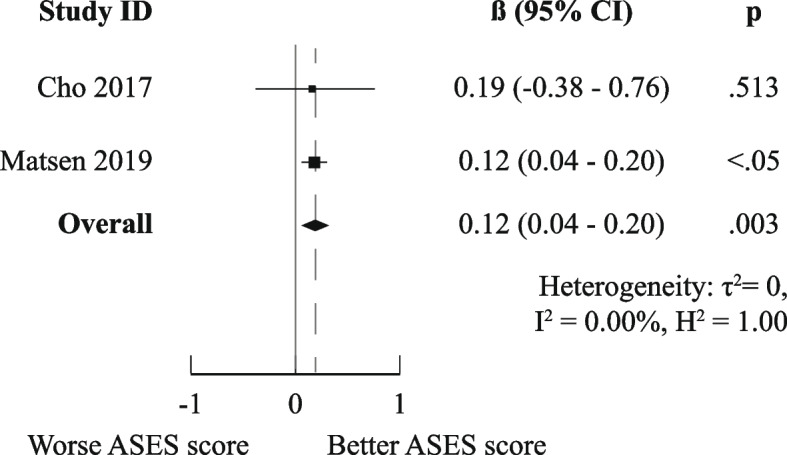
Meta-analysis results for prognostic factor "age" on raw ASES scores, medium-short term

**Fig. 4:**
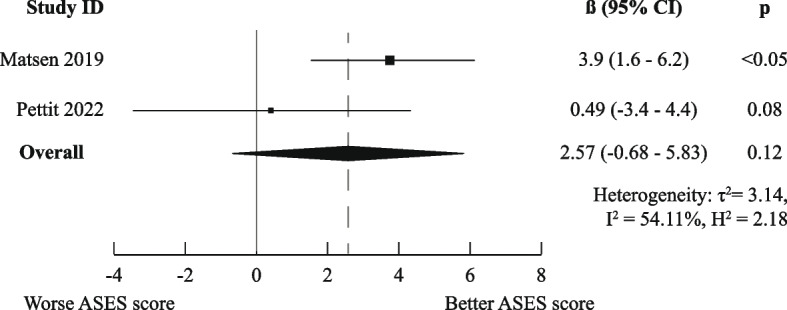
Meta-analysis results for prognostic factor "gender" on raw ASES scores, medium-long term

**Fig. 5:**
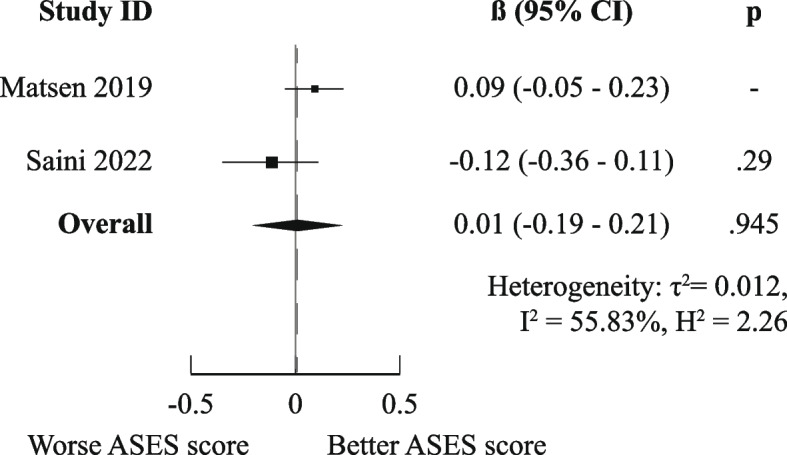
Meta-analysis results for prognostic factor "age" on raw ASES scores, medium-long term

**Fig. 6:**
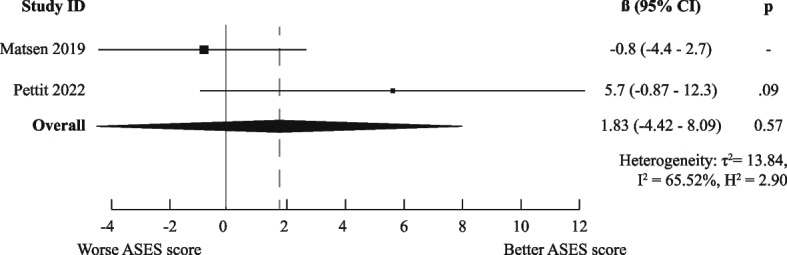
Meta-analysis results for prognostic factor "Walch A2 vs A1" on raw ASES scores, medium-long term

**Fig. 7:**
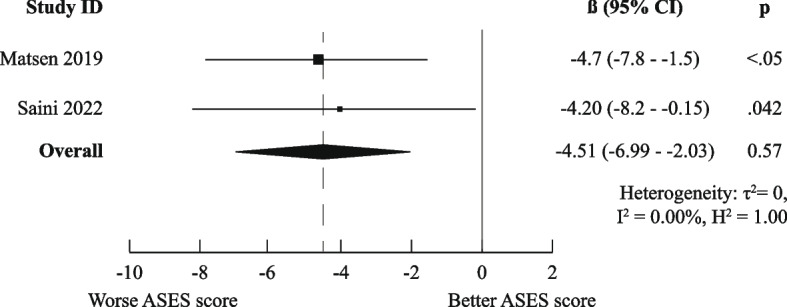
Meta-analysis results for prognostic factor "Previous surgery" on raw ASES scores, medium-long term

**Fig. 8:**
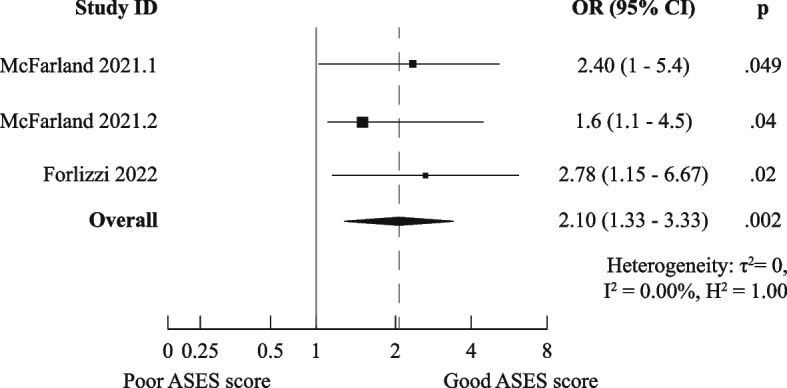
Meta-analysis results for prognostic factor "Previous surgery" on the odds of achieving poor ASES scores, medium-long term

Only the meta-analysis for medium-long term poor ASES scores indicated worse outcomes for patients who had undergone previous shoulder surgery (OR (95%CI) of 2.10 (1.33–3.33)). All other meta-analyses (for raw ASES scores) led to ORs and 95% CIs below the MCID, indicating neutral effects. The exact results are displayed in Figs. 3, 4, 5, 6, 7, and 8.

The planned sensitivity analyses did not alter the meaning of the pooled effect estimates and confidence intervals (Figs. A1–A2 in Appendix 2).

### Qualitative analysis and GRADE

**Fig. 9:**
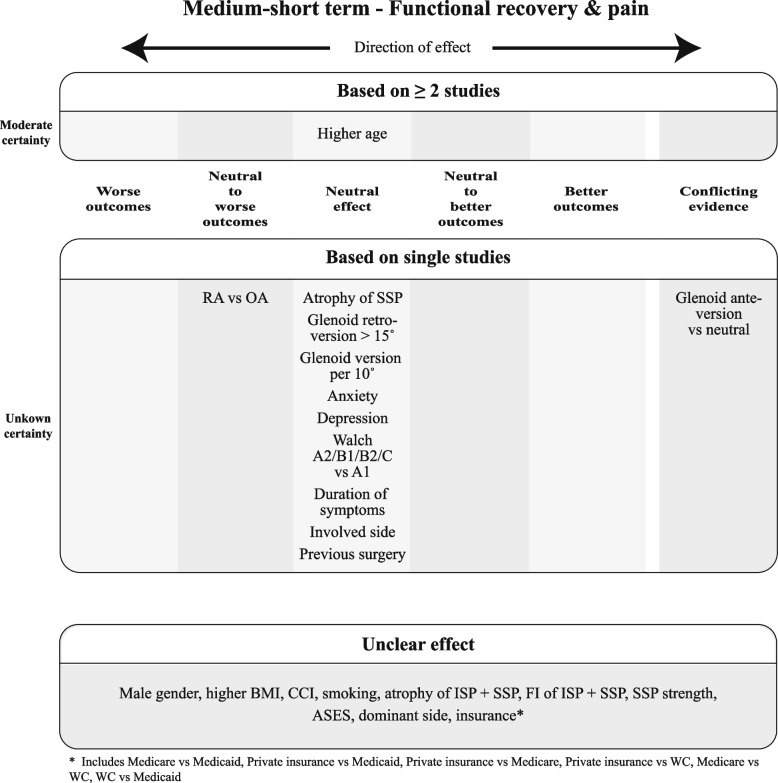
Summary of Findings for the domain "Functional Recovery & Pain", medium-short term

**Fig. 10:**
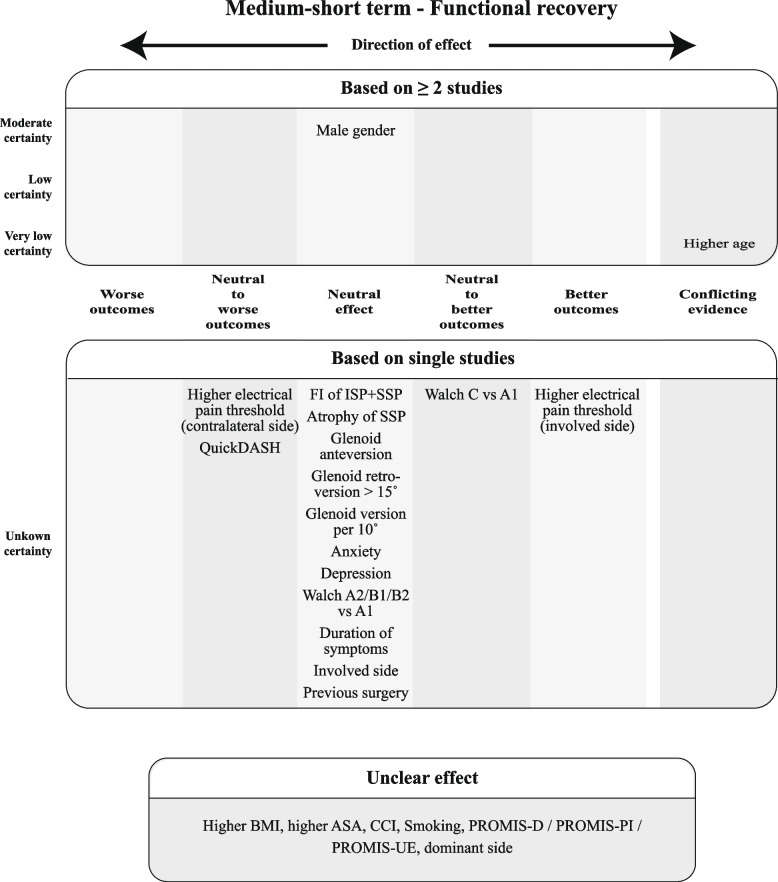
Summary of Findings for the domain "Functional Recovery", medium-short term

**Fig. 11:**
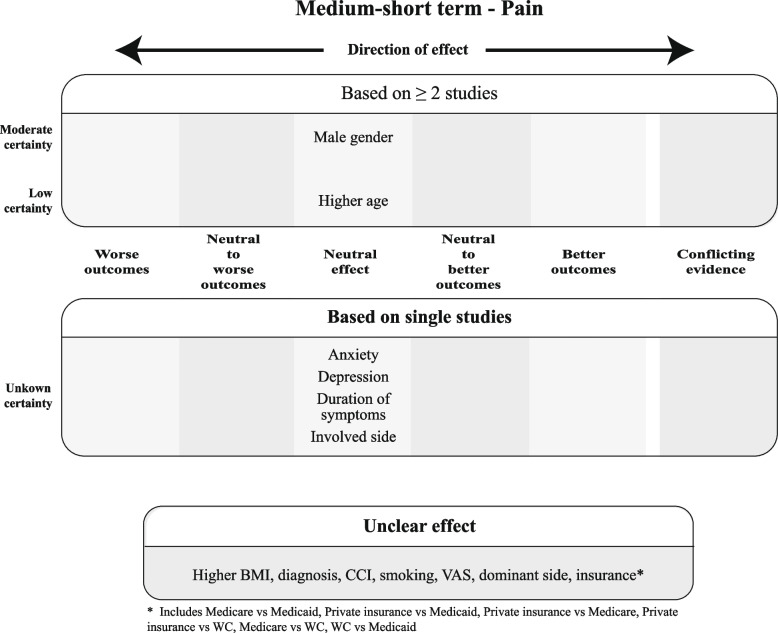
Summary of Findings for the domain "Pain", medium-short term

For each construct per time point, SoF Figs. 9, 10, and 11 summarize which prognostic factors were studied for medium-short-term outcomes, the direction of their effect and the certainty of the evidence according to GRADE. Figures A3–A8 (Appendix 2) depict the short-term, medium-long, and long-term outcomes. For the outcomes for which meta-analysis was possible, the direction and certainty were determined by combining that pooled result with the results from the additional studies that could not be meta-analyzed.

As the SoF figures show, no factor investigated in at least two studies showed distinct better or worse outcomes, but at most neutral to better or neutral to worse outcomes with varying degrees of certainty. In total, only nine factors showed better or worse outcomes, all examined in single studies with thus unknown certainty. The majority of factors were found to be either neutral or unclear in direction of effect based on the MCID.

For more details, Tables A3–A17 in Appendix 1 show all results from the individual studies, ordered per prognostic factor. It is clear that the effect estimates were not always reported for all variables included in the analyses.

Table A18 in Appendix 1 provides additional details on which domains the evidence was rated down or up.

## Discussion

The results of our review suggest that the strongest independent prognostic factors for better functional and pain-related outcomes after SA are “OA vs other diagnosis,” “private insurance,” and “higher electrical pain threshold on the operative side” and for worse outcomes “number of patient-reported allergies,” “back pain,” “living alone,” “CTA vs OA,” “diabetes,” and “greater preoperative range of motion in external rotation.” The evidence for these strongest predictors is of unknown certainty. Since they were only examined in single studies, using the GRADE system was not possible.

Prognostic factors that were examined in at least two studies and predicted neutral to better outcomes are “male gender” (low certainty). Neutral to worse outcomes were predicted by “depression” and “preoperative opioid use” (moderate certainty), “preoperative ASES scores,” “surgery on the dominant side,” and “previous surgery” (low certainty) and “male gender” (very low certainty).

Our results support those of recent systematic reviews that focused on a single predictor and used the results of univariable analyses. For example, both Mirghaderi et al. [53] and Ardebol et al. [54] also concluded that SA patients with previous shoulder surgery had worse postoperative outcomes than those without previous surgery. Where Ardebol et al. [54] only studied patients with massive rotator cuff tears (RCT) without glenohumeral arthritis, our included studies mainly focused on patients with osteoarthritis [36, 44, 46], thereby strengthening the evidence for this prognostic factor. In addition, Al-Mohrej et al. [55] found similar results to ours for preoperative opioid use: patients with preoperative opioid use had worse outcomes after shoulder surgery (including but not limited to SA) than patients without. Interestingly, in the included studies, the preoperative scores were worse for patients with preoperative opioid use as well, suggesting that maybe worse postoperative outcomes were a result of already having worse preoperative scores. However, the studies included in our current review [20, 27] used multivariable analyses that adjusted for preoperative scores and still found neutral to worse effects.

Interestingly, for the majority of reported factors, the direction of effect was unclear or neutral. This does not necessarily mean that these factors predict no effect at all; for the “unclear” factors, it was simply impossible to extract sufficient data to determine the most likely direction. For the “neutral” factors, some studies did report results that were statistically significantly different from predicting no effect, but the effect estimate and 95% CI failed to reach the MCID.

Only a few factors predicted distinct better or worse outcomes on their own when adjusted for other factors. These were reported only in single studies and most originated from studies using dichotomized endpoints with high contrasts. For example, Forlizzi et al. [27] compared patients scoring within the top 25th percentile of ASES scores with those scoring within the bottom 25th percentile, and Werner et al. [50] used the cut-off of having reached the substantial clinical benefit (SCB, in most instances larger than the MCID). Using smaller contrasts could still be relevant to patients and could lead to different and/or smaller effects.

### Strengths and limitations

An obvious limitation of our study is that we could not provide pooled estimates for many combinations of prognostic factors and outcomes despite the large number of studies we included. Two mechanisms were responsible for this. Firstly, studies used many different ways to analyze the same PROMs, resulting in important methodological heterogeneity. Secondly, the sheer amount of incomplete reporting and selective reporting in prognostic factor studies was a major problem, as has been noted before by other authors [56, 57]. The grouping of individual study results in Appendix 1 shows that for prognostic factors “higher age,” “male gender,” “anxiety,” and “dominant side,” additional meta-analyses would have been possible if only all results from the multivariable analysis were reported.

We can also highlight several strengths of this study.

Firstly, we did not limit ourselves to a predefined set of prognostic factors to include in the review, allowing us to compile all evidence from multivariable analyses currently available on this topic.

Secondly, although we could only perform a few meta-analyses, we have achieved a rigorous qualitative synthesis by using clear, transparent definitions to determine the direction of effect. Most systematic reviews without meta-analysis simply list the individual study results, while our approach enabled us to summarize the results.

Thirdly, we incorporated all combinations of prognostic factors and outcomes of the individual studies into our tables, whether the effect estimates were reported or not. This ensures transparency for which combinations information is missing, especially if only “*p* > 0.05” was reported.

Lastly, we applied the GRADE system to our qualitative analysis. While GRADE is designed foremost to grade quantitatively pooled estimates, it is no less important to state the overall certainty of available evidence in qualitative data synthesis. This also enabled us to take incomplete and selective reporting into account by downgrading the certainty of the evidence on the domain “publication bias” when present.

## Conclusion

Our systematic review has provided the first overview of current knowledge on the independent predictive effect of patient-related prognostic factors on functional recovery and pain-related outcomes after SA.

Based on the current body of evidence, a limited number of factors have a clinically relevant independent predictive effect, with an unknown certainty of evidence for predicting better or worse outcomes and mostly (very) low certainty of evidence for predicting neutral to better or neutral to worse outcomes. Promising factors for use in daily practice are a diagnosis of OA, multiple types of comorbidities, gender, insurance, living alone, multiple measures of preoperative status (e.g., ASES score, depression, opioid use, previous surgery, ROM), and surgery on the dominant side. However, further research is certainly necessary to confirm or nuance these results.

We strongly encourage future prognostic factor studies to report their complete findings and use more homogeneous outcomes. This will enable future research to refine the results of our first overview with more pooled results and clearer levels of certainty.

## Supplementary Information

Additional file 1: Appendix 1: Supplementary tables.

Additional file 2: Appendix 2: Supplementary figures.

## Data Availability

The datasets generated and analyzed during this systematic review are available from the corresponding author (BH) on reasonable request.
