# Supplementary material for: Patient-related prognostic factors for function and pain after shoulder arthroplasty: a systematic review
Source: Syst Rev. 2024 Nov 22;13:286. doi: 10.1186/s13643-024-02694-y (PMC11583791; doi:10.1186/s13643-024-02694-y)
Supplement: Supplementary file 1 — Additional file 1: Appendix 1: Supplementary tables. [file 13643_2024_2694_MOESM1_ESM.pdf]

Appendix 1 - Supplementary tables

Table A1 – Search strategy for PubMed

#	Query
**#1**	("Arthroplasty, Replacement"[Mesh] AND "Shoulder Joint"[Mesh])
**#2**	"Arthroplasty, Replacement, Shoulder"[Mesh]
**#3**	shoulder replacement*[tiab]
**#4**	shoulder arthroplast*[tiab]
**#5**	#1 OR #2 OR #3 OR #4
**#6**	"Osteoarthritis"[Mesh]
**#7**	"Arthritis"[Mesh]
**#8**	osteoarthrit*[tiab] OR osteo-arthrit*[tiab] OR arthrit*[tiab] OR arthropath*[tiab]
**9**	#6 OR #7 OR #8
**10**	"Patient Reported Outcome Measures"[Mesh]
**11**	"Visual Analog Scale"[Mesh]
**12**	"Range of Motion, Articular"[Mesh]
**13**	patient reported outcome*[tiab] OR PROM[tiab] OR PROMS[tiab] OR Oxford Shoulder Score[tiab] OR OSS[tiab] OR Quick DASH[tiab] OR quickdash[tiab] OR Shoulder Pain Disability Index[tiab] OR SPADI[tiab] OR Western Ontario[tiab] OR WOSI[tiab] OR WOOS[tiab] OR Shoulder Disability Questionnaire*[tiab] OR SDQ[tiab] OR Society Standardized Shoulder Assessment Form*[tiab] OR SSSAF[tiab] OR American Shoulder and Elbow Surgeons Standardized Shoulder Assessment Form*[tiab] OR ASES[tiab] OR numeric pain rating scal*[tiab] OR numeric rating scal*[tiab] OR NPRS[tiab] OR visual analog scal*[tiab] OR VAS pain[tiab] OR range of motion[tiab] OR AROM[tiab]
**14**	#10 OR #11 OR #12 OR #13
**15**	#5 AND #9 AND #14
**16**	"Letter"[Publication Type] OR "Editorial"[Publication Type] OR "News"[Publication Type] OR "Comment"[Publication Type] OR "Case Reports"[Publication Type] OR "Letter"[Title] OR "Editorial"[Title] OR "comment*"[Title] OR "case report"[Title]
**17**	#15 NOT #16

Table A2 – Minimal Clinically Important Differences (MCIDs)

Outcome measure	MCID
American Shoulder and Elbow Surgeons (ASES) score (1)	13.5
ASES function (2)	5.8*
ASES pain (2)	7.2*
Disabilities of Arm, Shoulder and Hand (DASH) score (3)	10.83
Oxford Shoulder Score (OSS) (4)	6.9
Patient-Reported Outcomes Measurement Information System – Depression (PROMIS-D) score (5)	3.9
Patient-Reported Outcomes Measurement Information System – Physical Functioning (PROMIS-PF) score (5)	1.7
Patient-Reported Outcomes Measurement Information System – Pain Interference (PROMIS-PI) score (5)	6.6
Patient-Reported Outcomes Measurement Information System – Upper Extremity (PROMIS-UE) score (5)	7
Penn Shoulder Score (PSS) (6)	12.1
Quick Disabilities of Arm, Shoulder and Hand (QuickDASH) score (7)	13.4
Single Assessment Numeric Evaluation (SANE) score (8)	18.1
Shoulder Pain and Disability Index (SPADI) score (9)	20.6
Simple Shoulder Test (STT) (10)	3
Visual Analogue Scale (VAS) pain score (11)	3.28
Western Ontario Osteoarthritis of the Shoulder (WOOS) % score (12)	10.2
WOOS raw score (recalculated from MCID WOOS % score)	193.8

* = Smallest Detectable Change used since no MCID was found in the literature

Table A3 - Individual results for prognostic factor ‘Male gender’

Study	n	n other factors	β/OR (95% CI)	p value	RoB	Forest plot
MALE GENDER ON FUNCTIONAL RECOVERY AND PAIN						
**MEDIUM-SHORT TERM**						
**ASES raw**						
Cho 2017	46	5	*±-8.41 (-21.95 - 5.13)***	.217	⊗	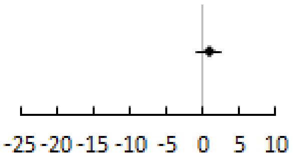
Matsen 2019	691	2	0.9 (-0.7 to 2.4)	-	⊗	
Wong 2017	117	6	-	>.05	⊖	
No data available from Lapner 2015						
**ASES improved**						
Chang 2022*	227	2	-	>.05	⊗	
**ASES %MPI**						
Matsen 2019	690	2	0.9 (-1.6 to 3.4)	-	⊗	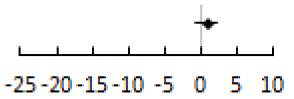
No data for WOOS% (Lapner 2015)						
**MEDIUM-LONG TERM**						
**ASES raw**						
Lansdown 2021	324	7	6 (-)	.023	⊗	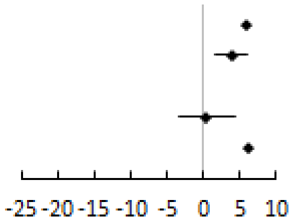
Matsen 2019	400	2	3.9 (1.6 to 6.2)	<.05	⊗	
Okoroha 2019	2364	5	-	<.01	⊗	
Pettit 2022	189	4	0.49 (-3.4 to 4.4)	.08	⊖	
Shields 2017	272	4	6.3 (-)	.01	⊗	
No data availabe from Dekker 2022, Moverman 2021, Patel 2019						
**ASES change**						
Friedman 2018	660	1	7.58 (5.27 to 9.89)	<.001	⊗	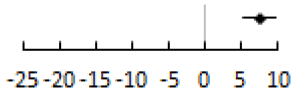
No data available from Patel 2019, Shields 2017						
**ASES %MPI**						
Matsen 2019	400	2	6.3 (0.7 to 12)	<.05	⊗	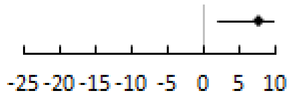
**ASES, poor score**						
No data available from McFarland 2021						
**ASES MOI**						
Polce 2021	105	3	0.87 (0.71 to 1.06)	.163	⊗	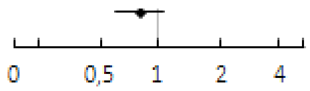
**ASES TE**						
Huber 2020	168	4	-0.02 (-0.09 to 0.04)	.518	⊖	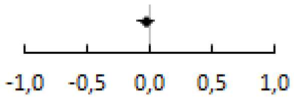
**SPADI change**						
Friedman 2018	660	1	-12.78 (-16.28 to -9.28)	<.001	⊗	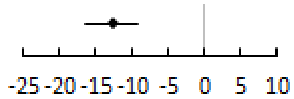
No data available from Patel 2019						
**LONG TERM**						
**ASES raw**						
Saini 2022	311	4	*±3.1 (-0.52 to 6.6)***	.094	⊖	

Table A3 - Individual results for prognostic factor ‘Male gender’ *(continued)*

Study	n	n other factors	β/OR (95% CI)	p value	RoB	Forest plot
**OSS raw**						
Edwards 2020	32	4	1.46 (-5.06 to 7.99)	.649	⊖	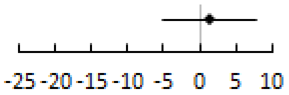
MALE GENDER ON FUNCTIONAL RECOVERY						
**SHORT TERM**						
**QuickDASH raw**						
Sayed-Noor 2018	63	6	-0.17 (-12 to 3)	.2	⊖	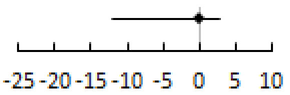
**MEDIUM-SHORT TERM**						
**ASES function raw**						
Wong 2017	117	6	-	.003	⊖	
**QuickDASH raw**						
Kadum 2018	63	6	.68 (-3.6 to 4.95)	.75	⊖	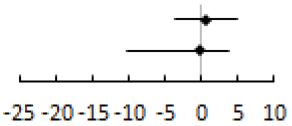
Sayed-Noor 2018	63	6	-0.13 (-10.4 to 3.8)	.35	⊖	
**SST100 raw**						
Matsen 2019	588	2	3.3 (0.4 to 6.3)	<.05	⊗	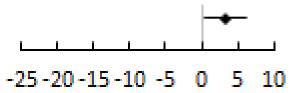
**SST %MPI**						
Matsen 2019	588	2	5 (0.2 to 9.9)	<.05	⊗	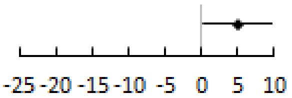
**SSV raw**						
Cho 2017	46	5	*±-2.04 (-10.3 - 6.3)***	.622	⊗	
No data for PROMIS-D / PROMIS-PI / PROMIS-UE (Franovic 2020)						
**MEDIUM-LONG TERM**						
**ASES function raw**						
Shields 2017	272	4	2.4 (-)	.003	⊗	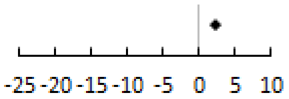
**SST raw**						
Okoroha 2019	2364	5	-	<.01	⊗	
No data available from Fehringer 2002, Green 2020, Patel 2019						
**SST change**						
Friedman 2018	660	1	1.41 (1.07 to 1.75)	<.001	⊗	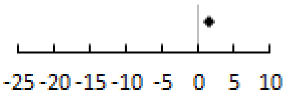
No data available from Green 2020, Patel 2019						
**SST100 raw**						
Matsen 2019	574	2	4.3 (1.1 to 7.4)	<.05	⊗	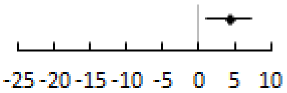
**SST100 %MPI**						
Matsen 2019	574	2	6.8 (2.4 to 11.3)	<.05	⊗	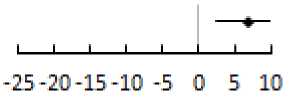

Table A3 - Individual results for prognostic factor 'Male gender' *(continued)*

Study	n	n other factors	β/OR (95% CI)	p value	RoB	Forest plot
No data for ASES function change (Shields 2017), DASH / DASH change (Green 2020), SANE raw (Dekker 2022, Moverman 2021), QuickDASH raw (Dekker 2022)						
**LONG TERM**						
**QuickDASH raw**						
Edwards 2020	32	4	-2.29 (-6.56 to 11.99)	.745	⊖	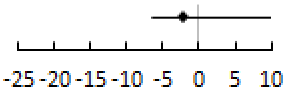
MALE GENDER ON PAIN						
**MEDIUM-SHORT TERM**						
**ASES pain raw**						
Wong 2017	17	6	-	>.05	⊖	
**VAS pain raw**						
Cho 2017	46	5	*±0.61 (-1.01 - 2.23)***	.45	⊗	
Wong 2017	117	6	-	>.05	⊖	
**MEDIUM-LONG TERM**						
No data for VAS pain (Green 2020, Moverman 2021, Shields 2017), VAS pain change (Green 2020, Patel 2019, Shields 2017)						

* Chang 2022 reported a simple and complex model, only the complex model was extracted for this table

** Cho 2017 and Saini 2022 did not specify whether 'male' or 'female' was the reference category, so the direction of effect cannot be interpreted

Table A4 - Individual results for prognostic factor ‘Higher age’

HIGHER AGE ON FUNCTIONAL RECOVERY AND PAIN

MEDIUM-SHORT TERM

ASES raw

Study	n	n other factors	β/OR (95% CI)	p value	RoB
Cho 2017	46	5	0.19 (-0.38 to 0.76)	.513	⊗
Matsen 2019	691	2	0.12 (0.04 to 0.2)*	<.05	⊗
Wong 2017	117	6	-	>.05	⊖
No data available from Lapner 2015					

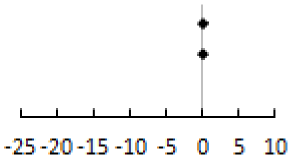

ASES %MPI

Study	n	n other factors	β/OR (95% CI)	p value	RoB
Matsen 2019	691	2	0.16 (0.02 to 0.29)*	<.05	⊗

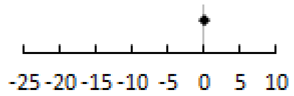

No data for WOOS% (Lapner 2015)

MEDIUM-LONG TERM

ASES raw

Study	n	n other factors	β/OR (95% CI)	p value	RoB
Matsen 2019	400	2	0.09 (-0.05 to 0.23)*	-	⊗
Patel 2019	2364	5	-	.069	⊗
Pettit 2022	197	4	-0.28 (-0.6 to 0.04)	.08	⊖
Saini 2022	311	4	-0.12 (-0.36 to 0.11)	.29	⊖
No data availabe from Dekker 2022, Lansdown 2021, Okoroha 2019, Shields 2017					

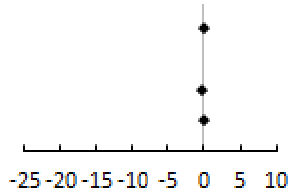

ASES change

Study	n	n other factors	β/OR (95% CI)	p value	RoB
Friedman 2018	660	1	0.19 (0.04 to 0.34)	.011	⊗
Patel 2019	1135	4	-	.87	⊗
No data available from Shields 2017					

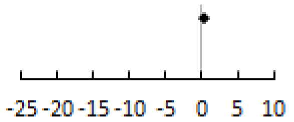

ASES %MPI

Study	n	n other factors	β/OR (95% CI)	p value	RoB
Matsen 2019	400	2	0.11 (-0.22 to 0.44)*	-	⊗

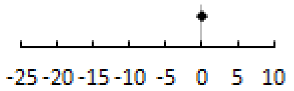

ASES, poor score

No data available from McFarland 2021

ASES, poor improvement

Study	n	n other factors	β/OR (95% CI)	p value	RoB
Chang 2022**	227	2	-	>.05	⊗

ASES TE

Study	n	n other factors	β/OR (95% CI)	p value	RoB
Huber 2020	168	4	0.02 (-0.05 to 0.1)Ƭ	.546	⊖
Huber 2020	168	4	0.09 (-0.01 to 0.2)Ŧ	.087	⊖

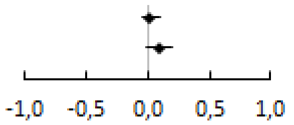

SPADI raw

Study	n	n other factors	β/OR (95% CI)	p value	RoB
Patel 2019	1135	4	-	.051	⊗

SPADI change

Study	n	n other factors	β/OR (95% CI)	p value	RoB
Friedman 2018	660	1	-0.29 (-0.46 to 0.07)	.02	⊗
Patel 2019	1135	4	-	.223	⊗

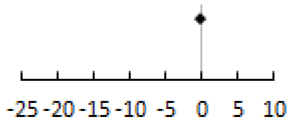

Table A4 - Individual results for prognostic factor ‘Higher age’ *(continued)*

Study	n	n other factors	β/OR (95% CI)	p value	RoB	Forest plot
**LONG TERM**						
**OSS raw**						
Edwards 2020	32	4	-0.41 (-0.9 to 0.08)	.094	⊖	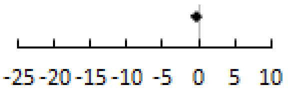
HIGHER AGE ON FUNCTIONAL RECOVERY						
**SHORT TERM**						
**QuickDASH raw**						
Sayed-Noor 2018	63	6	-0.17 (-0.73 to -0.15)	.2	⊖	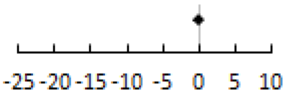
**MEDIUM-SHORT TERM**						
**ASES function raw**						
Wong 2017	117	6	-	>.05	⊖	
**QuickDASH raw**						
Kadum 2018	63	6	0.15 (-0.14 to 0.44)	.29	⊖	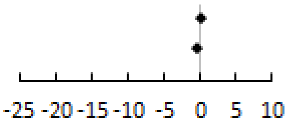
Sayed-Noor 2018	63	6	-0.48 (-0.92 to -0.04)	.03	⊖	
**SST100 raw**						
Matsen 2019	588	2	0.07 (-0.08 to 0.23)*	-	⊗	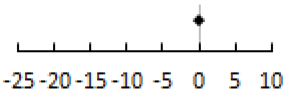
**SST100 %MPI**						
Matsen 2019	588	2	0.07 (-0.17 to 0.32)*	-	⊗	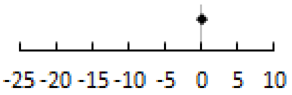
**SSV raw**						
Cho 2017	46	5	0.13 (-0.22 to 0.48)	.465	⊗	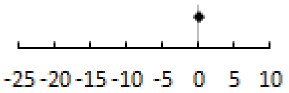
No data for PROMIS-UE / PROMIS-PI / PROMIS-D (Franovic 2020)						
**MEDIUM-LONG TERM**						
**SST raw**						
Patel 2019	1135	4	-	.313	⊗	
No data available from Fehringer 2002, Green 2020, Okoroha 2019						
**SST change**						
Friedman 2018	660	1	0.01 (-0.02 to 0.03)	.611	⊗	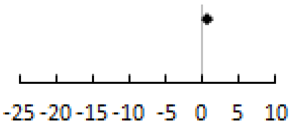
Patel 2019	1135	4	-	.708	⊗	
No data available from Green 2020						
**SST100 raw**						
Matsen 2019	574	2	0.18 (0.03 to 0.34)*	<.05	⊗	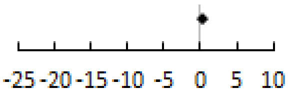
**SST100 %MPI**						
Matsen 2019	574	2	0.2 (-0.02 to 0.43)*	-	⊗	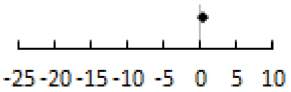

Table A4 - Individual results for prognostic factor ‘Higher age’ *(continued)*

Study	n	n other factors	β/OR (95% CI)	p value	RoB	Forest plot
No data for ASES ADL / ASES ADL change (Shields 2017), QuickDASH (Dekker 2022), DASH / DASH change (Green 2020), SANE (Dekker 2020), SST % improvement (Fehringer 2002), SSV / SSV change (Shields 2017)						
**LONG TERM**						
**QuickDASH raw**						
Edwards 2020	32	4	0.86 (-0.21 to 1.93)	.11	⊖	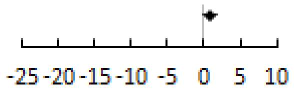
HIGHER AGE ON PAIN						
**MEDIUM-SHORT TERM**						
**ASES pain raw**						
Wong 2017	117	6	-	>.05	⊖	
**VAS pain raw**						
Cho 2017	46	5	-0.03 (-0.1 to 0.04)	.398	⊗	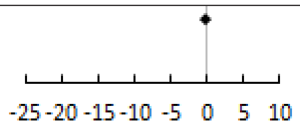
Wong 2017	117	6	-	>.05	⊖	
**MEDIUM-LONG TERM**						
**VAS pain raw**						
Patel 2019	1135	4	-	.03	⊗	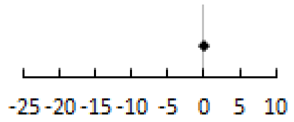
Shields 2017	272	4	-0.03 (-)	.04	⊗	
No data available from Green 2020						
**VAS pain change**						
Patel 2019	1135	4	-	.564	⊗	
No data available from Green 2020, Shields 2017						

* Recalculated to reflect change per year of age; Matsen 2019 reported per 10 years of age

** Chang 2022 reported a simple and complex model, only the complex model was extracted for this table

† Age 65-70 compared to age < 65

‡ Age > 70 compared to age < 65

⊤ Age 70-80 compared to age < 70

⊤̄ Age 80+ compared to age < 70

Table A5 - Individual results for prognostic factor ‘Higher BMI’

Study	n	n other factors	β/OR (95% CI)	p value	RoB	Forest plot
HIGHER BMI ON FUNCTIONAL RECOVERY AND PAIN						
**MEDIUM-SHORT TERM**						
**ASES raw**						
Wong 2017	117	6	-	>.05	⊖	
**MEDIUM-LONG TERM**						
**ASES, poor score**						
No data available from McFarland 2021						
No data for ASES raw (Lansdown 2021, Okoroha 2019, Patel 2019, Shields 2017), ASES change (Patel 2019, Shields 2017), SPADI / SPADI change / UCLA / UCLA change (Patel 2019)						
HIGHER BMI ON FUNCTIONAL RECOVERY						
**MEDIUM-SHORT TERM**						
**ASES function raw**						
Wong 2017	117	6	-	>.05	⊖	
Outcomes without data						
PROMIS-D / PROMIS-PI / PROMIS-UE (Franovic 2020)						
**MEDIUM-LONG TERM**						
No data for ASES ADL (Shields 2017), SST raw (Okoroha 2019, Patel 2019), SST change (Patel 2019), SSV (Shields 2017)						
HIGHER BMI ON PAIN						
**MEDIUM-SHORT TERM**						
**ASES pain raw**						
Wong 2017	117	6	-	>.05	⊖	
**VAS pain raw**						
Wong 2017	117	6	-	>.05	⊖	
**MEDIUM-LONG TERM**						
No data for VAS pain (Patel 2019, Shields 2017)						

Table A6 - Individual results for prognostic factor ‘ASA score’

Study	n	n other factors	β/OR (95% CI)	p value	RoB	Forest plot
ASA SCORE ON FUNCTIONAL RECOVERY AND PAIN						
**MEDIUM-LONG TERM**						
**ASES TE**						
Huber 2020	168	4	-0.02 (-0.16 to 0.12)†	.79	⊖	
Huber 2020	168	4	-0.09 (-0.21 to 0.03)‡	.157	⊖	
Huber 2020	168	4	-0.16 (-0.28 to -0.03)ǂ	.013	⊖	
ASA SCORE ON FUNCTIONAL RECOVERY						
**MEDIUM-SHORT TERM**						
No data for PROMIS-D / PROMIS-PI / PROMIS-UE (Kohan 2020)						

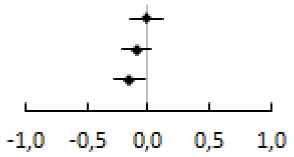

† ASA II compared to ASA I
‡ ASA III compared to ASA I
ǂ ASA ≥ IV compared to ASA I

Table A7 - Individual results for prognostic factor ‘Diagnosis’

Study	n	n other factors	β/OR (95% CI)	p value	RoB	Forest plot
DIAGNOSIS ON FUNCTIONAL RECOVERY AND PAIN						
**MEDIUM-SHORT TERM**						
**Diagnosis: RA compared to OA**						
**ASES raw**						
Lapner 2015	62	8	-	>.05	⊗	
**WOOS%**						
Lapner 2015	62	8	-16.8 (-30.3 to -3.2)	.017	⊗	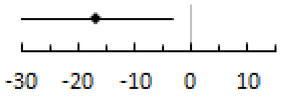
**MEDIUM-LONG TERM**						
**Diagnosis: OA compared to CTA**						
**ASES raw**						
Saini 2022	311	4	9.3 (5.4 to 13.1)	<.01	⊖	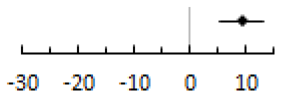
*No data availabe from Moverman 2021**						
**Diagnosis: RCA compared to degenerative joint disease**						
**ASES, poor score**						
Carducci 2019	137	3	1.79 (0.66 to 4.75)	-	⊗	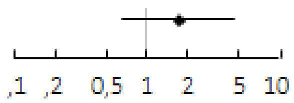
**ASES, poor improvement**						
Carducci 2019	137	3	1.43 (0.44 to 4.53)	-	⊗	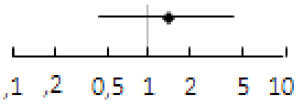
**Diagnosis: RCT compared to degenerative joint disease**						
**ASES, poor score**						
Carducci 2019	137	3	2.21 (0.75 to 6.37)	-	⊗	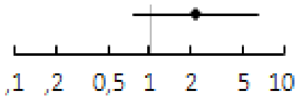
**ASES, poor improvement**						
Carducci 2019	137	3	2.42 (0.67 to 8.83)	-	⊗	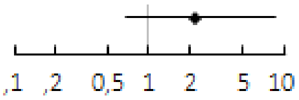
No data for ASES change / UCLA / UCLA change / SPADI / SPADI change (Patel 2019)						
**Diagnosis: OA compared to other diagnosis**						
**ASES, poor improvement**						
Forlizzi 2022	162	8	0.17 (0.03 to 0.84)†	.03	⊖	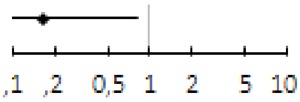
**Diagnosis: RCA compared to other diagnosis**						
**ASES, poor improvement**						
Forllizzi 2022	162	8	0.71 (0.16 to 3.33)†	.69	⊖	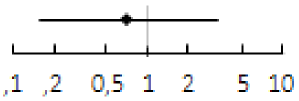

Table A7 - Individual results for prognostic factor ‘Diagnosis’ *(continued)*

Study	n	n other factors	β/OR (95% CI)	p value	RoB	Forest plot
**LONG TERM**						
**Diagnosis: CTA compared to OA**						
**ASES SCB**						
Werner 2016	490	3	0.14 (0.07 to 0.3)	<.001	⊗	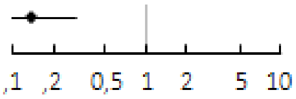
DIAGNOSIS ON FUNCTIONAL RECOVERY						
**MEDIUM-LONG TERM**						
No data for SANE (Moverman 2021), SST (Okoroha 2019, Patel 2019), SST change (Patel 2019)						
DIAGNOSIS ON PAIN						
**MEDIUM-SHORT TERM**						
No data for VAS pain raw (Moverman 2021, Patel 2019), VAS pain change (Patel 2019)						

* Reference category unclear

† Inverse of reported odds ratio and confidence interval (reported OR was for top 25% of ASES scores)

Table A8 - Individual results for prognostic factor ‘Comorbidities’

Study	n	n other factors	β/OR (95% CI)	p value	RoB	Forest plot
COMORBIDITIES ON FUNCTIONAL RECOVERY AND PAIN						
**MEDIUM-SHORT TERM**						
**Charlson Comorbidity Index**						
**ASES raw**						
Wong 2017	117	6	-	>.05	⊖	
**Smoking**						
**ASES raw**						
Wong 2017	117	6	-	>.05	⊖	
**MEDIUM-LONG TERM**						
**Diabetes Mellitus**						
**ASES MOI**						
Polce 2021	105	3	0.74 (0.53 to 1.05)	.091	⊗	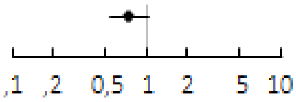
**Comorbidity back pain**						
**ASES SCB**						
Werner 2016	490	3	0.42 (0.24 to 0.71)	.002	⊗	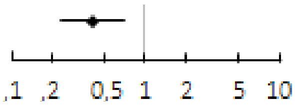
**Presence of functional somatic disorder**						
**ASES raw**						
Moverman 2021	480	6	-9.75 (-13.61 to -5.89)	<.001	⊗	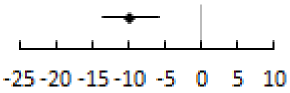
**Number of patient-reported allergies**						
**ASES top score**						
Forlizzi 2022	162	8	0.83 (0.71 to 0.97)	.02	⊖	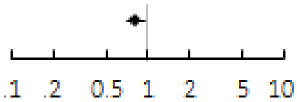
No data for ASES raw (Moverman 2021)						
**Charlson Comorbidity Index**						
No data for ASES raw (Lansdown 2021)						
**Comorbidities**						
No data for ASES raw (McFarland 2021)						
**Smoking**						
No data for ASES raw (McFarland 2021)						

Table A8 - Individual results for prognostic factor ‘Comorbidities’ *(continued)*

Study	n	n other factors	β/OR (95% CI)	p value	RoB	Forest plot
COMORBIDITIES ON FUNCTIONAL RECOVERY						
**MEDIUM-SHORT TERM**						
**Charlson Comorbidity Index**						
**ASES function raw**						
Wong 2017	117	6	-	>.05		
**Smoking**						
**ASES function raw**						
Wong 2017	117	6	-	>.05		
**MEDIUM-LONG TERM**						
**Diabetes Mellitus**						
**SANE MOI**						
Polce 2021	105	1	0.66 (0.47 to 0.93)	.02		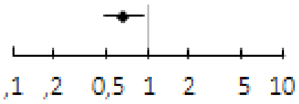
**Presence of functional somatic disorder**						
**SANE raw**						
Moverman 2021	480	6	-7.63 (-12.12 to -3.15)	.001		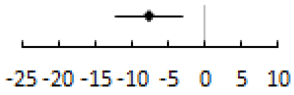
**Number of comorbidities**						
No data for DASH / DASH change / SST / SST change (Green 2020)						
**Number of patient-reported allergies**						
No data for SANE (Moverman 2021)						
COMORBIDITIES ON PAIN						
**MEDIUM-SHORT TERM**						
**Charlson Comorbidity Index**						
**ASES pain raw**						
Wong 2017	117	6	-	>.05		
**VAS pain raw**						
Wong 2017	117	6	-	>.05		
**Smoking**						
**ASES pain raw**						
Wong 2017	117	6	-	>.05		
**VAS pain raw**						
Wong 2017	117	6	-	>.05		

Table A8 - Individual results for prognostic factor ‘Comorbidities’ *(continued)*

Study	n	n other factors	β/OR (95% CI)	p value	RoB	Forest plot
**MEDIUM-LONG TERM**						
**Presence of functional somatic disorder**						
**VAS pain raw**						
Moverman 2021	480	6	1.13 (0.72 to 1.53)	<.001		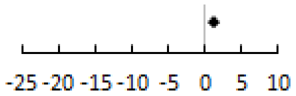
**Number of comorbidities**						
No data for VAS pain / VAS pain change (Green 2020)						
**Number of patient-reported allergies**						
No data for VAS pain (Moverman 2021)						

Table A9 - Individual results for prognostic factor ‘Rotator cuff status’

Study	n	n other factors	β/OR (95% CI)	p value	RoB	Forest plot
ROTATOR CUFF STATUS ON FUNCTIONAL RECOVERY AND PAIN						
**MEDIUM-SHORT TERM**						
**Preoperative supraspinatus occupation area**						
**WOOS%**						
Lapner 2015	62	8	0.27 (0.06 to 0.47)	.013	⊗	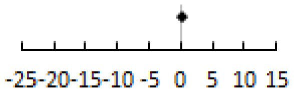
No data for ASES raw (Lapner 2015)						
**Preoperative infraspinatus and supraspinatus Warner grade + Preoperative infraspinatus and supraspinatus fatty infiltration + Preoperative infraspinatus and supraspinatus Goutallier grade + Preoperative supraspinatus strength**						
No data for ASES raw / WOOS% (Lapner 2015)						
**MEDIUM-LONG TERM**						
**Preoperative supraspinatus occupation area**						
No data availabe for ASES raw (Lapner 2015)						
**LONG TERM**						
**Preoperative supraspinatus Warner grade**						
**OSS raw**						
Edwards 2020	32	4	3.99 (-5.45 to 13.44)	.393	⊖	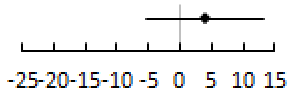
**Rotator cuff muscles fatty infiltration**						
**OSS raw**						
Edwards 2020	32	4	-2.76 (-9.55 to 4.03)	.411	⊖	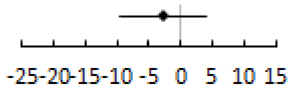
**Tendinopathy grade**						
**OSS raw**						
Edwards 2020	32	4	3.97 (-0.77 to 8.71)	.097	⊖	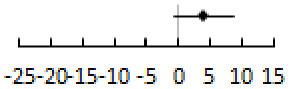
ROTATOR CUFF STATUS ON FUNCTIONAL RECOVERY						
**SHORT TERM**						
**Preoperative infraspinatus Warner grade**						
**QuickDASH raw**						
Sayed-Noor 2018	63	6	-0.09 (-14 to 7)	.48	⊖	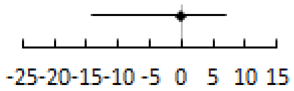

Table A9 - Individual results for prognostic factor ‘Rotator cuff status’ *(continued)*

Study	n	n other factors	β/OR (95% CI)	p value	RoB	Forest plot
**Preoperative infraspinatus Goutallier grade**						
**QuickDASH raw**						
Sayed-Noor 2018	63	6	0.17 (-5 to 9)	.60	⊖	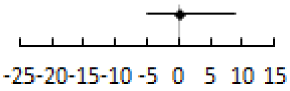
**Preoperative supraspinatus Warner grade**						
**QuickDASH raw**						
Sayed-Noor 2018	63	6	-0.04 (-6.3 to 5)	.80	⊖	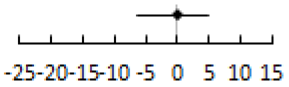
**Preoperative supraspinatus Goutallier grade**						
**QuickDASH raw**						
Sayed-Noor 2018	63	6	0.32 (-0.68 to 10)	.08	⊖	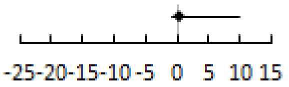
**MEDIUM-SHORT TERM**						
**Preoperative infraspinatus Warner grade**						
**QuickDASH raw**						
Sayed-Noor 2018	63	6	-0.09 (-14 to 7)	.54	⊖	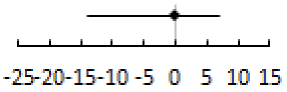
**Preoperative infraspinatus Goutallier grade**						
**QuickDASH raw**						
Sayed-Noor 2018	63	6	0.22 (-3 to 10.5)	.28	⊖	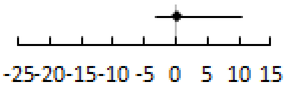
**Preoperative supraspinatus Warner grade**						
**QuickDASH raw**						
Sayed-Noor 2018	63	6	-0.02 (-5.7 to 5.2)	.92	⊖	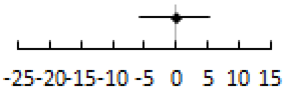
**Preoperative supraspinatus Goutallier grade**						
**QuickDASH raw**						
Sayed-Noor 2018	63	6	0.01 (-0.059 to 5.9)	.98	⊖	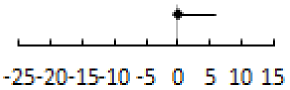
**MEDIUM-LONG TERM**						
**Tear limited to supraspinatus + infraspinatus**						
**SST 30%MPI**						
Somerson 2016	42	3	∞ (2.01 to ∞)	.02	⊗	
**LONG TERM**						
**Preoperative supraspinatus Warner grade**						
**QuickDASH raw**						
Edwards 2020	32	4	2.06 (-12.79 to 16.92)	.778	⊖	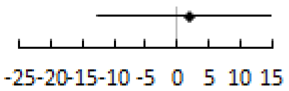

Table A9 - Individual results for prognostic factor ‘Rotator cuff status’ *(continued)*

Study	n	n other factors	β/OR (95% CI)	p value	RoB	Forest plot
**Rotator cuff muscles fatty infiltration**						
**QuickDASH raw**						
Edwards 2020	32	4	-5.62 (-26.28 to 15.04)	.581	⊖	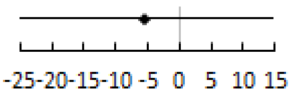
**Tendinopathy grade**						
**QuickDASH raw**						
Edwards 2020	32	4	-9.21 (-19.58 to 1.15)	.079	⊖	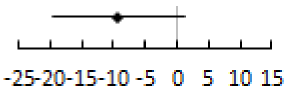

∞ Infinity

Table A10 - Individual results for prognostic factor 'Glenohumeral characteristics'

Study	n	n other factors	β/OR (95% CI)	p value	RoB	Forest plot
GLENOHUMERAL CHARACTERISTICS ON FUNCTIONAL RECOVERY AND PAIN						
**MEDIUM-SHORT TERM**						
**Preoperative glenoid retroversion (per 10°)**						
**ASES raw**						
Matsen 2019	168	2	0.5 (-2 to 2.9)	-	⊗	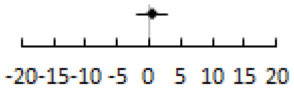
**ASES %MPI**						
Matsen 2019	168	2	1.5 (-12.3 to 15.2)	-	⊗	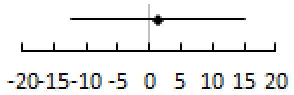
**Preoperative glenoid anteversion compared to neutral**						
**ASES raw**						
Matsen 2019	691	2	2.1 (-17.8 to 21.9)	-	⊗	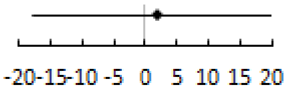
**ASES %MPI**						
Matsen 2019	691	2	6.9 (-30.4 - 44.1)	-	⊗	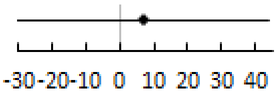
**Preoperative glenoid retroversion >15° compared to neutral**						
**ASES raw**						
Matsen 2019	691	2	1 (-2.2 to 4.1)	-	⊗	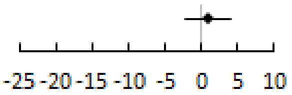
**ASES %MPI**						
Matsen 2019	691	2	2.4 (-3.6 to 8.4)	-	⊗	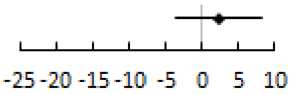
**MEDIUM-LONG TERM**						
**Preoperative glenoid retroversion (per 10°)**						
**ASES raw**						
Matsen 2019	187	2	2.8 (0.1 to 5.5)	<.05	⊗	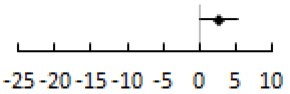
No data available from Dekker 2022						
**ASES %MPI**						
Matsen 2019	187	2	14 (-9.1 to 37.1)	-	⊗	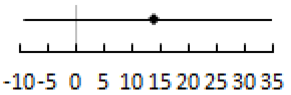
**Preoperative glenoid anteversion compared to neutral**						
**ASES raw**						
Matsen 2019	400	2	-0.4 (-34.6 to 33.9)	-	⊗	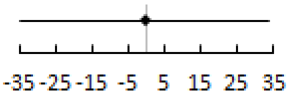
**ASES %MPI**						
Matsen 2019	400	2	3.4 (-95.4 to 102.3)	-	⊗	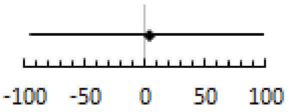

Table A10 - Individual results for prognostic factor ‘Glenohumeral characteristics’ *(continued)*

Study	n	n other factors	β/OR (95% CI)	p value	RoB	Forest plot
**Preoperative glenoid retroversion >15° compared to neutral**						
**ASES raw**						
Matsen 2019	400	2	3.4 (-0.8 to 7.5)	-	⊗	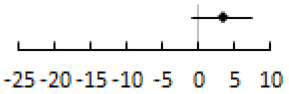
**ASES %MPI**						
Matsen 2019	400	2	6.7 (-5.3 to 18.8)	-	⊗	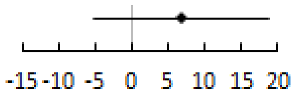
**Glenoid inclination (degrees)**						
No data for ASES raw (Dekker 2022)						
GLENOHUMERAL CHARACTERISTICS ON FUNCTIONAL RECOVERY						
**MEDIUM-SHORT TERM**						
**Preoperative glenoid retroversion (per 10°)**						
**SST100 raw**						
Matsen 2019	347	2	1.5 (-0.8 to 3.7)	-	⊗	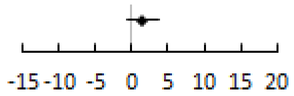
**SST100 %MPI**						
Matsen 2019	347	2	5.4 (-3.6 to 14.4)	-	⊗	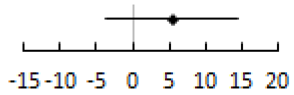
**Preoperative glenoid anteversion compared to neutral**						
**SST100 raw**						
Matsen 2019	588	2	3.9 (-6.7 to 14.5)	-	⊗	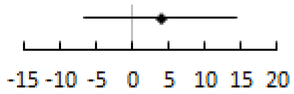
**SST100 %MPI**						
Matsen 2019	588	2	4.1 (-9.9 to 18.2)	-	⊗	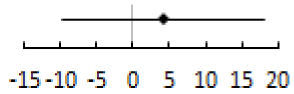
**Preoperative glenoid retroversion >15° compared to neutral**						
**SST100 raw**						
Matsen 2019	588	2	3.8 (-0.6 to 8.2)	-	⊗	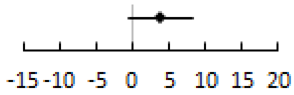
**SST100 %MPI**						
Matsen 2019	588	2	4.6 (-1.2 to 10.5)	-	⊗	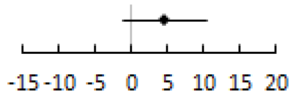
**MEDIUM-LONG TERM**						
**Preoperative glenoid retroversion (per 10°)**						
**SST100 raw**						
Matsen 2019	334	2	1.8 (-0.5 to 4.2)	-	⊗	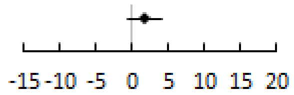
**SST100 %MPI**						
Matsen 2019	334	2	8.3 (-0.6 to 17.3)	-	⊗	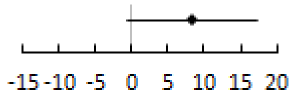

No data for SANE raw / QuickDASH raw (Dekker 2022)

Table A10 - Individual results for prognostic factor ‘Glenohumeral characteristics’ *(continued)*

Study	n	n other factors	β/OR (95% CI)	p value	RoB	Forest plot
**Preoperative glenoid anteversion compared to neutral**						
**SST100 raw**						
Matsen 2019	574	2	1.1 (-9.5 to 11.6)	-	⊗	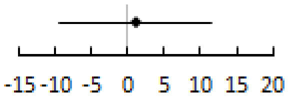
**SST100 %MPI**						
Matsen 2019	574	2	-0.2 (-13.9 to 13.4)	-	⊗	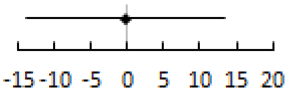
**Preoperative glenoid retroversion >15° compared to neutral**						
**SST100 raw**						
Matsen 2019	574	2	4.3 (-0.4 to 9.1)	-	⊗	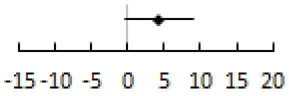
**SST100 %MPI**						
Matsen 2019	574	2	6 (-0.2 to 12.1)	-	⊗	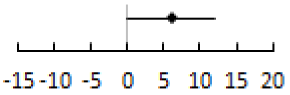
**Glenoid inclination**						
No data for SANE raw / QuickDASH raw (Dekker 2022)						

Table A11 - Individual results for prognostic factor ‘Depression and Anxiety’

Study	n	n other factors	β/OR (95% CI)	p value	RoB	Forest plot
DEPRESSION AND ANXIETY ON FUNCTIONAL RECOVERY AND PAIN						
**MEDIUM-SHORT TERM**						
**HADS Depression**						
**ASES raw**						
Cho 2017	46	5	0.14 (-2.03 to 2.31)	.897	⊗	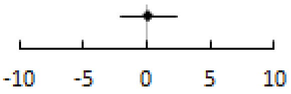
**HADS Anxiety**						
**ASES raw**						
Cho 2017	46	5	0.79 (-1.32 to 2.89)	.454	⊗	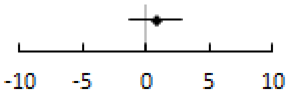
**MEDIUM-LONG TERM**						
**Depression**						
**ASES raw**						
Kohan 2020	111	2	-2.5 (-10.1 to 5.2)	.53	⊗	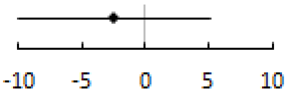
**ASES change**						
Werner 2017	616	?	-	.018	⊗	
**ASES top score**						
Forlizzi 2022	162	8	0.39 (0.16 to 0.99)	.046	⊖	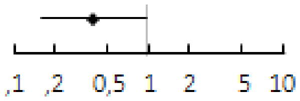
**WOOS raw**						
Kohan 2020	111	2	40.1 (-98.7 to 178.8)	.57	⊗	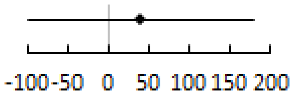
**Anxiety**						
**ASES raw**						
Kohan 2020	111	2	-3.6 (-10.6 to 3.4)	.31	⊗	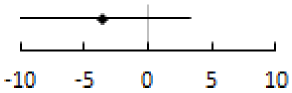
No data available from Moverman 2021						
**WOOS raw**						
Kohan 2020	111	2	74.7 (-50.9 to 200.3)	.24	⊗	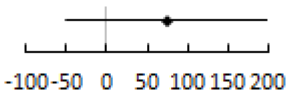
DEPRESSION AND ANXIETY ON FUNCTIONAL RECOVERY						
**MEDIUM-SHORT TERM**						
**HADS Depression**						
**SSV raw**						
Cho 2017	46	5	0.53 (-0.8 to 1.86)	.426	⊗	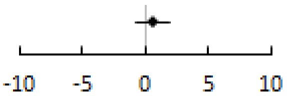

Table A11 - Individual results for prognostic factor ‘Depression and Anxiety’ *(continued)*

Study	n	n other factors	β/OR (95% CI)	p value	RoB	Forest plot
**HADS Anxiety**						
**SSV raw**						
Cho 2017	46	5	-0.01 (-1.3 to 1.28)	.982	⊗	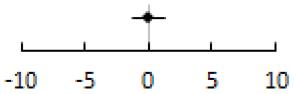
**MEDIUM-LONG TERM**						
**Depression**						
**PROMIS-UE raw**						
Kohan 2020	111	2	2.7 (-1.2 to 6.7)	.17	⊗	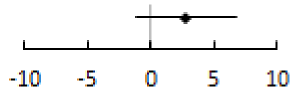
**PROMIS-PF raw**						
Kohan 2020	111	2	1.6(-2.4 to 5.5)	.43	⊗	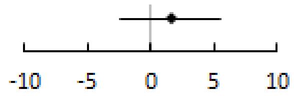
**PROMIS-PI raw**						
Kohan 2020	111	2	-0.8 (-4.5 to 2.9)	.68	⊗	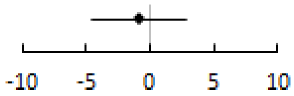
**Anxiety**						
**PROMIS-UE raw**						
Kohan 2020	111		-4.6 (-8.2 to -0.9)	.02	⊗	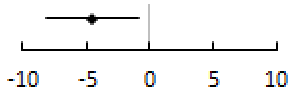
**PROMIS-PF raw**						
Kohan 2020	111		-3.3 (-6.8 to 0.3)	.07	⊗	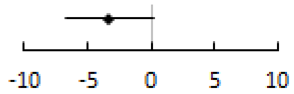
**PROMIS-PI raw**						
Kohan 2020	111		4.3 (0.9 to 7.7)	.01	⊗	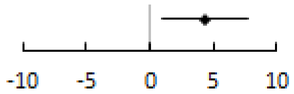
No data for SANE raw (Moverman 2021)						
**Anxiety/depression**						
No data for DASH raw / SST raw / DASH change / SST change (Green 2020)						

DEPRESSION AND ANXIETY ON PAIN

Study	n	n other factors	β/OR (95% CI)	p value	RoB	Forest plot
**MEDIUM-SHORT TERM**						
**HADS Depression**						
**VAS pain raw**						
Cho 2017	46	5	-0.02 (-0.28 to 0.24)	.899	⊗	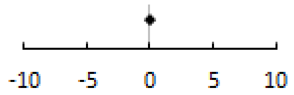
**HADS Anxiety**						
**VAS pain raw**						
Cho 2017	46	5	-0.01 (-0.36 to 0.14)	.382	⊗	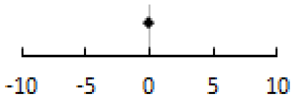

Table A11 - Individual results for prognostic factor ‘Depression and Anxiety’ *(continued)*

Study	n	n other factors	β/OR (95% CI)	p value	RoB	Forest plot
**MEDIUM-LONG TERM**						
**Depression**						
**VAS pain raw**						
Kohan 2020	111	2	0.5 (-0.4 to 1.4)	.29	⊗	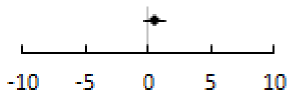
**Anxiety**						
**VAS pain raw**						
Kohan 2020	111	2	0.4 (-0.5 to 1.2)	.39	⊗	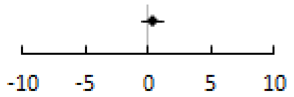
No data available from Moverman 2021						
**Anxiety/depression**						
No data available for VAS pain / VAS pain change (Green 2020)						

Table A12 - Individual results for prognostic factor ‘Expectations’

Study	n	n other factors	β/OR (95% CI)	p value	RoB	Forest plot
EXPECTATIONS ON FUNCTIONAL RECOVERY AND PAIN						
**MEDIUM-LONG TERM**						
**Number of very important expectations**						
**ASES raw**						
Swarup 2017	67	?	1.53 (-)	.007		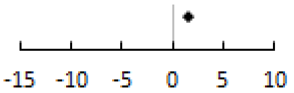
No data available from Rauck 2018						
**ASES change**						
Swarup 2017	67	?	1.13 (-)	.037		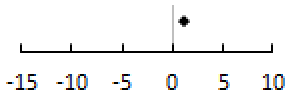
**Expectation ‘Relieving nighttime pain’**						
**ASES raw**						
Rauck 2018	137	?	7 (-)	.048		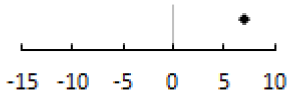
No data available from Rauck 2018						
EXPECTATIONS ON FUNCTIONAL RECOVERY						
**MEDIUM-LONG TERM**						
**Mean preoperative expectation score**						
**DASH raw**						
Green 2020	176	8	-10 (-15 to -3)	.007		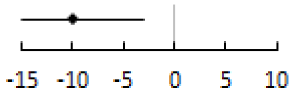
**DASH change**						
Green 2020	176	8	-10 (-15 to -3)	.004		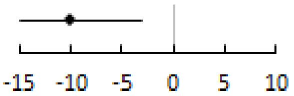
**SST raw**						
Green 2020	176	8	0.7 (-0.5 to 1.9)	.29		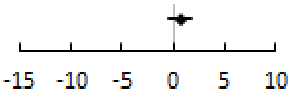
**SST change**						
Green 2020	176	8	0.7 (-0.5 to 1.9)	.265		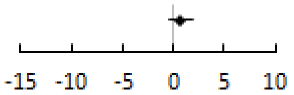
**Expectation ‘Improving non-overhead sports**						
**SAS raw**						
Rauck 2018	137	?	2.8 (-)	.02		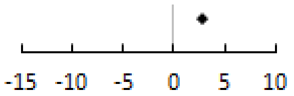
**Confidence in achieving outcomes**						
**PSS raw**						
Styron 2015	436	8	2.65 (0.14 to 5.16)	.039		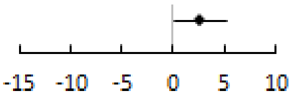

Table A12 - Individual results for prognostic factor ‘Expectations’ *(continued)*

Study	n	n other factors	β/OR (95% CI)	p value	RoB	Forest plot
			EXPECTATIONS ON PAIN			
**MEDIUM-LONG TERM**						
**Number of very important expectations**						
**VAS pain raw**						
Swarup 2017	67	?	-0.16 (-)*	.01	⊗	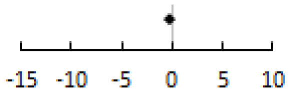
No data available from Rauck 2018						
**VAS pain change**						
Swarup 2017	67	?	-0.15 (-)*	.02	⊗	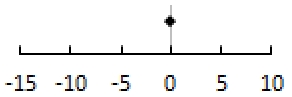
**Expectation ‘Relieving nighttime pain’**						
**VAS pain raw**						
Rauck 2018	137	?	-0.59 (-)*	.047	⊗	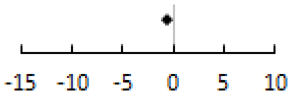
**VAS pain change**						
Rauck 2018	137	?	-0.61 (-)*	.039	⊗	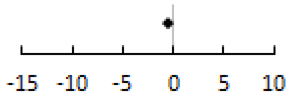
**Mean preoperative expectation score**						
**VAS pain raw**						
Green 2020	176	8	-0.4 (-1.3 to 0.6)*	.453	⊗	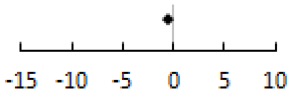
**VAS pain change**						
Green 2020	176	8	-0.4 (-1.3 to 0.6)*	.452	⊗	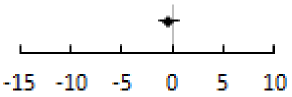
**Confidence in achieving outcomes**						
**PSS pain raw**						
Styron 2015	436	8	1.99 (0.17 to 3.82)	.033	⊖	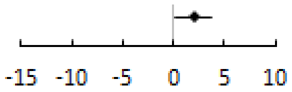

* Recalculated to reflect VAS on 0 - 10 scale instead of 0 - 100 as reported in studies

Table A13 - Individual results for prognostic factor ‘Classification systems’

Study	n	n other factors	β/OR (95% CI)	p value	RoB	Forest plot
CLASSIFICATION SYSTEMS ON FUNCTIONAL RECOVERY AND PAIN						
**MEDIUM-SHORT TERM**						
**Walch A2 compared to A1**						
**ASES raw**						
Matsen 2019	380	2	0 (-3.2 to 3.3)	-	⊗	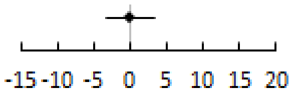
**ASES %MPI**						
Matsen 2019	380	2	-0.9 (-6.4 to 4.6)	-	⊗	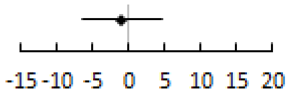
**Walch B1 compared to A1**						
**ASES raw**						
Matsen 2019	380	2	1.9 (-1.4 to 5.1)	-	⊗	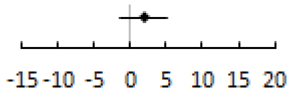
**ASES %MPI**						
Matsen 2019	380	2	4 (-1.5 to 9.5)	-	⊗	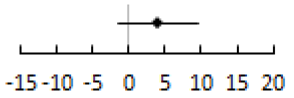
**Walch B2 compared to A1**						
**ASES raw**						
Matsen 2019	380	2	-1.2 (-4.3 to 1.9)	-	⊗	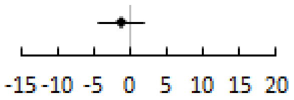
**ASES %MPI**						
Matsen 2019	380	2	-2.7 (-8 to 2.6)	-	⊗	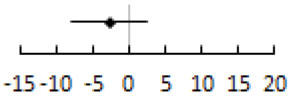
**Walch C compared to A1**						
**ASES raw**						
Matsen 2019	380	2	2.4 (-5.5 to 10.2)	-	⊗	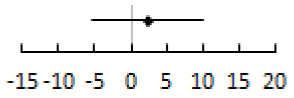
**ASES %MPI**						
Matsen 2019	380	2	2.6 (-10.8 to 15.9)	-	⊗	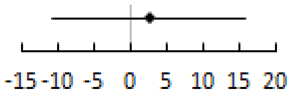
**MEDIUM-LONG TERM**						
**Walch A2 compared to A1**						
**ASES raw**						
Matsen 2019	380	2	-0.8 (-4.4 to 2.7)	-	⊗	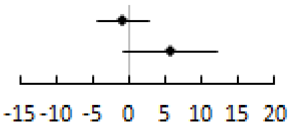
Pettit 2022	197	4	5.7 (-0.87 to 12.3)*	.09	⊖	
**ASES %MPI**						
Matsen 2019	380	2	-0.7 (-9.6 to 8.2)	-	⊗	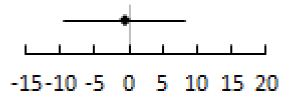
**Walch B1 compared to A1**						
**ASES raw**						
Matsen 2019	380	2	0.1 (-3.3 to 3.6)	-	⊗	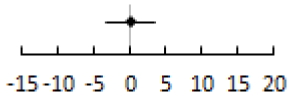

Table A13 - Individual results for prognostic factor ‘Classification systems’ *(continued)*

Study	n	n other factors	β/OR (95% CI)	p value	RoB	Forest plot
**ASES %MPI**						
Matsen 2019	380	2	1.4 (-7.3 to 10.1)	-	⊗	
**Walch B1 compared to A2**						
**ASES raw**						
Pettit 2022	197	4	-2.3 (-12.3 to 7.4)	.64	⊖	
**Walch B2 compared to A1**						
**ASES raw**						
Matsen 2019	380	2	0.5 (-2.9 to 3.9)	-	⊗	
**ASES %MPI**						
Matsen 2019	380	2	-0.5 (-9 to 8)	-	⊗	
**Walch B2 compared to A2**						
**ASES raw**						
Pettit 2022	197	4	0.43 (-6.1 to 6.9)	.9	⊖	
**Walch B3 compared to A2**						
**ASES raw**						
Pettit 2022	197	4	2.5 (-4.3 to 9.2)	.47	⊖	
**Walch C compared to A1**						
**ASES raw**						
Matsen 2019	380	2	2.8 (-6.7 to 12.4)	-	⊗	
**ASES %MPI**						
Matsen 2019	380	2	4.3 (-19.7 to 28.2)	-	⊗	
**Concentric wear (Walch A1/A2) compared to eccentric wear (B1/B2/B3/D)**						
**ASES raw**						
Kohan 2020	111	2	-3.5 (-9.5 to 2.5)	.26	⊗	
**WOOS raw**						
Kohan 2020	111	2	62.7 (-46 to 171.4)	.26	⊗	
**Walch classification**						
**ASES raw**						
Dekker 2022	168	6	-	>.05	⊗	
**Hamada grade 3 compared to Hamada grade 2**						
**ASES TE**						
Huber 2020	168	4	0.05 (-0.03 to 0.14)	.299	⊖	

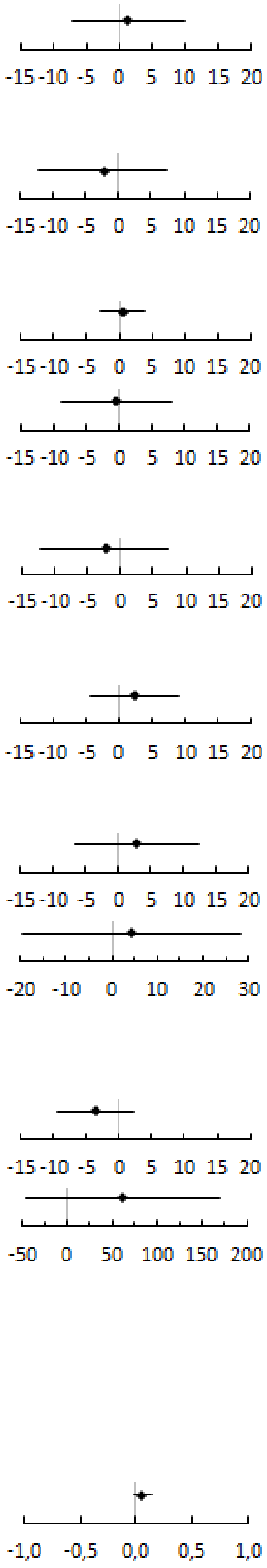

Table A13 - Individual results for prognostic factor ‘Classification systems’ *(continued)*

Study	n	n other factors	β/OR (95% CI)	p value	RoB	Forest plot
**Hamada grade ≥4 compared to Hamada grade 2**						
**ASES TE**						
Huber 2020	168	4	0.08 (0 to 0.15)	.042	⊖	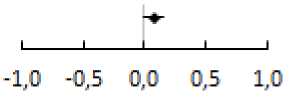
CLASSIFICATION SYSTEMS ON FUNCTIONAL RECOVERY						
**MEDIUM-SHORT TERM**						
**Walch A2 compared to A1**						
**SST100 raw**						
Matsen 2019	588	2	-0.8 (-5 to 3.4)	-	⊗	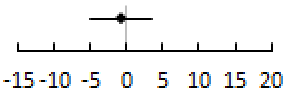
**SST100 %MPI**						
Matsen 2019	588	2	1.3 (-5.6 to 8.1)	-	⊗	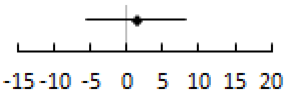
**Walch B1 compared to A1**						
**SST100 raw**						
Matsen 2019	588	2	1.2 (-3.5 to 5.9)	-	⊗	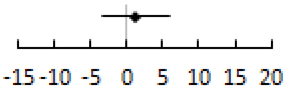
**SST100 %MPI**						
Matsen 2019	588	2	5.3 (-2.4 to 13)	-	⊗	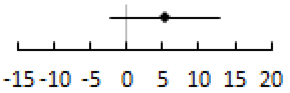
**Walch B2 compared to A1**						
**SST100 raw**						
Matsen 2019	588	2	1.5 (-3.2 to 6.2)	-	⊗	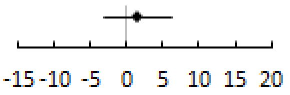
**SST100 %MPI**						
Matsen 2019	588	2	5 (-2.7 to 12.6)	-	⊗	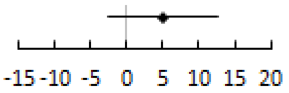
**Walch C compared to A1**						
**SST100 raw**						
Matsen 2019	588	2	5.1 (-12.3 to 22.5)	-	⊗	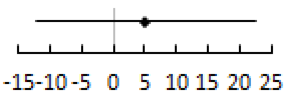
**SST100 %MPI**						
Matsen 2019	588	2	10.9 (-17.5 to 39.3)	-	⊗	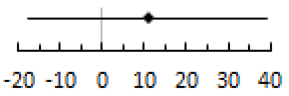
**MEDIUM-LONG TERM**						
**Walch A2 compared to A1**						
**SST100 raw**						
Matsen 2019	574	2	0.4 (-4.3 to 5)	-	⊗	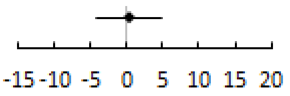
**SST100 %MPI**						
Matsen 2019	574	2	1.7 (-4.9 to 8.3)	-	⊗	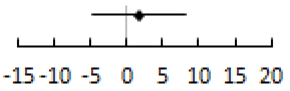

Table A13 - Individual results for prognostic factor ‘Classification systems’ *(continued)*

Study	n	n other factors	β/OR (95% CI)	p value	RoB	Forest plot
**Walch B1 compared to A1**						
**SST100 raw**						
Matsen 2019	574	2	2.5 (-2.7 to 7.7)	-	⊗	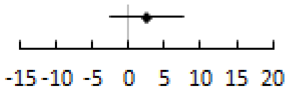
**SST100 %MPI**						
Matsen 2019	574	2	5.2 (-2.2 to 12.5)	-	⊗	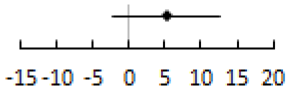
**Walch B2 compared to A1**						
**SST100 raw**						
Matsen 2019	574	2	1.1 (-4 to 6.3)	-	⊗	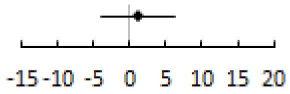
**SST100 %MPI**						
Matsen 2019	574	2	3.5 (-3.8 to 10.9)	-	⊗	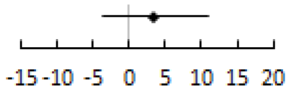
**Walch C compared to A1**						
**SST100 raw**						
Matsen 2019	574	2	7.2 (-11.2 to 25..5)	-	⊗	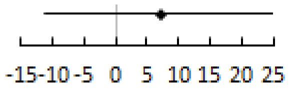
**SST100 %MPI**						
Matsen 2019	574	2	11.7 (-14.3 to 37.8)	-	⊗	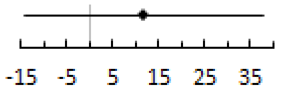
**Concentric wear (Walch A1/A2) compared to eccentric wear (B1/ B2/B3/D)**						
**PROMIS-UE raw**						
Kohan 2020	111	2	-3 (-6.1 to 0.1)	.06	⊗	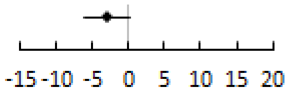
**PROMIS-PF raw**						
Kohan 2020	111	2	-4.2 (-7.3 to -1.1)	<.01	⊗	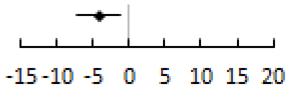
**PROMIS-PI raw**						
Kohan 2020	111	2	3.2 (0.3 to 6.1)	.03	⊗	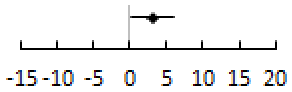
**Walch classification**						
**ASES raw**						
Dekker 2022	168	6	-	>.05	⊗	
CLASSIFICATION SYSTEMS ON PAIN						
**MEDIUM-LONG TERM**						
**Concentric wear (Walch A1/A2) compared to eccentric wear (B1/ B2/B3/D)**						
**VAS pain raw**						
Kohan 2020	111	2	0.5 (-0.5 to 1.2)	.19	⊗	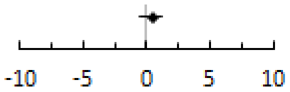

† Inverse of reported regression coefficient and confidence interval (reported estimates were for Walch A1 compared to A2)

Table A14 - Individual results for prognostic factor ‘Preoperative status’

Study	n	n other factors	β/OR (95% CI)	p value	RoB	Forest plot
PREOPERATIVE STATUS ON FUNCTIONAL RECOVERY AND PAIN						
**MEDIUM-SHORT TERM**						
**Preoperative ASES score**						
No data for ASES raw (Strotman 2020)						
**MEDIUM-LONG TERM**						
**Preoperative ASES score**						
**ASES raw**						
Pettit 2022	197	4	0.06 (-0.06 to 0.2)	.35	⊖	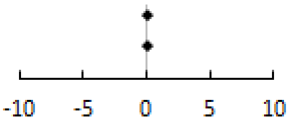
Saini 2022	311	4	0.14 (0.03 to 0.24)	.01	⊖	
No data available from Dekker 2022, Moverman 2021						
**ASES poor score**						
Carducci 2019	137	3	0.95 (0.72 to 1.24)	-	⊗	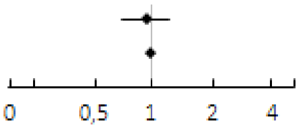
Forlizzi 2022	162	8	1 (0.96 to 1.01)*	.27	⊖	
**ASES poor improvement**						
Carducci 2019	137	3	2.96 (2.04 to 4.62)	-	⊗	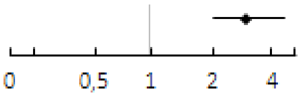
**ASES MCID**						
Werner 2016	490	1	0.96 (0.94 to 0.98)	<.001	⊗	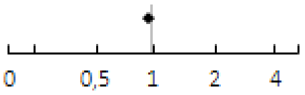
**ASES SCB**						
Werner 2016	490	3	0.91 (0.89 to 0.92)	<.001	⊗	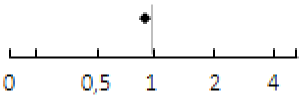
PREOPERATIVE STATUS ON FUNCTIONAL RECOVERY						
**SHORT TERM**						
**Preoperative QuickDASH score**						
**QuickDASH raw**						
Sayed-Noor 2018	63	6	0.3 (0.04 to 0.58)	.027	⊖	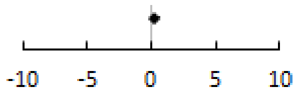
**MEDIUM-SHORT TERM**						
**Preoperative QuickDASH score**						
**QuickDASH raw**						
Kadum 2018	63	6	-0.02 (-0.17 to 0.14)	.8	⊖	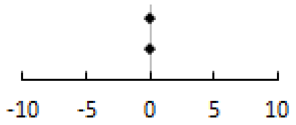
Sayed-Noor 2018	63	6	0.03 (-0.25 to 0.3)	.85	⊖	

Table A14 - Individual results for prognostic factor ‘Preoperative status’ *(continued)*

Study	n	n other factors	β/OR (95% CI)	p value	RoB	Forest plot
**Preoperative electrical pain threshold operative side**						
**QuickDASH raw**						
Kadum 2018	63	6	-2.2 (-3.1 to -1.3)	.0001	⊖	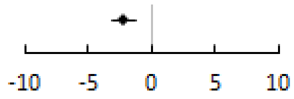
**Preoperative pain at rest**						
**QuickDASH raw**						
Kadum 2018	63	6	0.23 (0.07 to 0.39)	.007	⊖	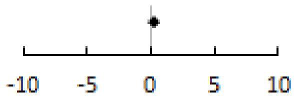
**Preoperative pain on exertion**						
**QuickDASH raw**						
Kadum 2018	63	6	0.05 (-0.07 to 0.17)	.43	⊖	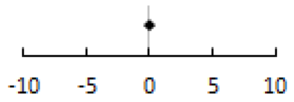
**Preoperative electrical pain threshold contralateral side**						
**QuickDASH raw**						
Kadum 2018	63	6	0.14 (-0.28 to 0.56)	.49	⊖	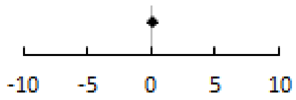
**Preoperative PROMIS-D score**<br>**Preoperative PROMIS-PI score**<br>**Preoperative PROMIS-UE score**						
No data available for PROMIS-D / PROMIS-PI / PROMIS-UE (Franovic 2020)						
**MEDIUM-LONG TERM**						
**Preoperative SST score**						
**SST raw**						
Chawla 2022	39	?	-	<.001	⊗	
No data available from Green 2020						
**SST 30% MPI**						
Somerson 2016	42	3	0.56 (0.17 to 1.04)	.068	⊗	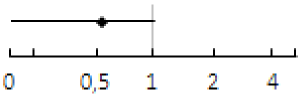
No data for SST change (Green 2020)						
**Preoperative active external rotation**						
**SST 30% MPI**						
Somerson 2016	42	3	0.71 (0.38 to 0.9)	<.001	⊗	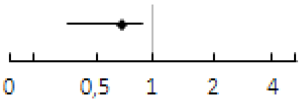
**Preoperative VAS pain score**						
**SST raw**						
Chawla 2022	39	?	-	>.05	⊗	
No data available from Green 2020						
**SST 30% MPI**						
Somerson 2016	42	3	1.02 (0.95 to 1.11)	.611		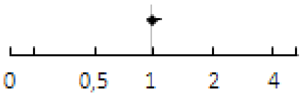
**Preoperative DASH score**						
No data for DASH raw / DASH change (Green 2020)						

Table A14 - Individual results for prognostic factor ‘Preoperative status’ *(continued)*

Study	n	n other factors	β/OR (95% CI)	p value	RoB	Forest plot
**Preoperative QuickDASH score**						
No data for QuickDASH raw (Dekker 2022)						
**Preoperative SANE score**						
No data for SANE raw (Dekker 2022, Moverman 2021)						
**Preoperative shoulder function (SST categorical)**						
No data for SST raw / SST change / SST % improvement (Fehringer 2002)						
PREOPERATIVE STATUS ON PAIN						
**MEDIUM-SHORT TERM**						
**Preoperative VAS pain score**						
No data for VAS pain raw (Strotman 2020)						
**MEDIUM-LONG TERM**						
**Preoperative VAS pain score**						
No data for VAS pain (Green 2020, Moverman 2021), VAS pain change (Green 2020)						

* Inverse of reported odds ratio and confidence interval (reported OR was for top 25% of ASES scores)

Table A15 - Individual results for prognostic factor ‘Sociodemographic factors’

Study	n	n other factors	β/OR (95% CI)	p value	RoB	Forest plot
SOCIODEMOGRAPHIC FACTORS ON FUNCTIONAL RECOVERY AND PAIN						
**MEDIUM-LONG TERM**						
**Living alone**						
**ASES SCB**						
Werner 2016	490	3	0.36 (0.19 to 0.69)	.002		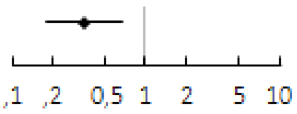
**College education**						
**Level of activity at work**						
**Marital status**						
No data for ASES poor score (McFarland 2021)						
**Home zip code median income**						
No data for ASES raw (Lansdown 2021)						
SOCIODEMOGRAPHIC FACTORS ON FUNCTIONAL RECOVERY						
**MEDIUM-LONG TERM**						
**College education**						
**Work status**						
No data for DASH / DASH change / SST / SST change (Green 2020)						
SOCIODEMOGRAPHIC FACTORS ON PAIN						
**MEDIUM-LONG TERM**						
**College education**						
**Work status**						
No data for VAS pain raw / VAS pain change (Green 2020)						

Table A16 - Individual results for prognostic factor ‘Symptoms and History’

Study	n	n other factors	β/OR (95% CI)	p value	RoB	Forest plot
SYMPTOMS AND HISTORY ON FUNCTIONAL RECOVERY AND PAIN						
**MEDIUM-SHORT TERM**						
**Duration of symptoms**						
**ASES raw**						
Cho 2017	46	5	0.02 (-0.03 to 0.06)	.531	⊗	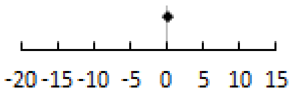
**Dominant side / hand dominance**						
**ASES raw**						
Wong 2017	117	6	-	>.05	⊖	
No data available from Lapner 2015						
No data for WOOS% (Lapner 2015)						
**Involved side**						
**ASES raw**						
Cho 2017	46	5	*±0.04 (-12.8 - 12.9)**	.995	⊗	
**Previous surgery**						
**ASES raw**						
Matsen 2019	691	2	-4.6 (-6.9 to -2.4)	<.05	⊗	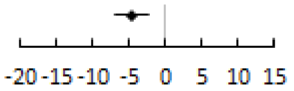
**ASES %MPI**						
Matsen 2019	691	2	-8.5 (-12.2 to -4.8)	<.05	⊗	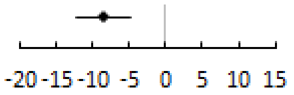
**MEDIUM-LONG TERM**						
**Dominant side**						
**ASES MOI**						
Polce 2021	105	3	0.83 (0.68 to 1.01)	.061	⊗	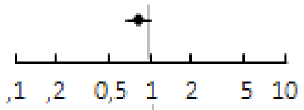
**ASES TE**						
Huber 2020	168	4	0.1 (-0.01 to 0.21)**	.07	⊖	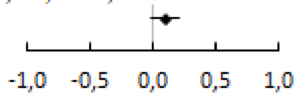
No data for ASES poor score (McFarland 2021)						
**Preoperative opioid use**						
**ASES poor score**						
Carducci 2019[†]	137	3	3.26 (1.08 to 10.07)	-	⊗	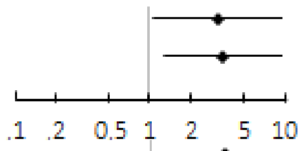
Forlizzi 2022	162	8	3.48 (1.32 to 11.11)[‡]	.01	⊖	
**ASES poor improvement**						
Carducci 2019[†]	137	3	3.38 (0.9 to 12.75)	-	⊗	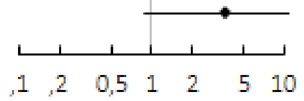

Table A16 - Individual results for prognostic factor ‘Symptoms and History’ *(continued)*

Study	n	n other factors	β/OR (95% CI)	p value	RoB	Forest plot
**Previous surgery**						
**ASES raw**						
Shields 2017	272	4	-9.5 (-)	<.001	⊗	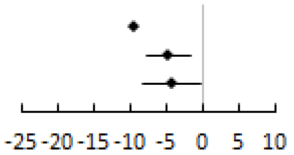
Matsen 2019	400	2	-4.7 (-7.8 to -1.5)	<.05	⊗	
Saini 2022	311	4	-4.2 (-8.2 to -0.15)	.042	⊖	
No data available from Okoroha 2019, Patel 2019						
**ASES change**						
Shields 2017	272	4	-7.9 (-)	.012	⊗	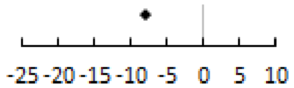
No data available from Patel 2019						
**ASES %MPI**						
Matsen 2019	40	2	-11.9 (-19.6 to -4.3)	-	⊗	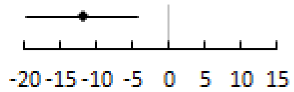
**ASES, poor score**						
McFarland 2021	19	9	2.4 (1 to 5.4)⊤	.049	⊗	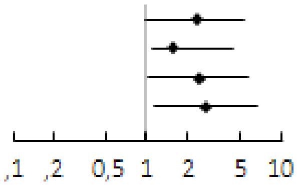
McFarland 2021	25	9	1.6 (1.1 to 4.5)╤	.04	⊗	
Carducci 2019[†]	137	3	2.46 (1.03 to 5.83)	-	⊗	
Forlizzi 2022	162	8	2.78 (1.15 to 6.67)[‡]	.02	⊖	
**ASES, poor improvement**						
Carducci 2019[†]	137	3	3.77 (1.34 to 11.23)	-	⊗	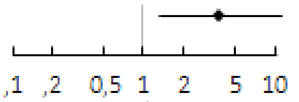
**ASES MOI**						
Polce 2021	105	3	0.83 (0.68 to 1.01)	.061	⊗	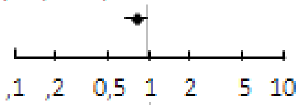
**ASES TE**						
Huber 2020	168	4	-0.1 (-0.21 to 0.01)	.07	⊖	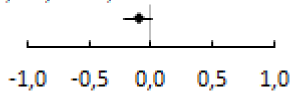
No data for SPADI raw / SPADI change / UCLA raw / UCLA change (Patel 2019)						
**Percentage subluxation**						
No data for ASES raw (Dekker 2022)						
SYMPTOMS AND HISTORY ON FUNCTIONAL RECOVERY						
**MEDIUM-SHORT TERM**						
**Duration of symptoms**						
**SSV raw**						
Cho 2017	46	5	-0.001 (-0.031 to 0.028)	.931	⊗	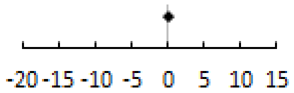
**Dominant side**						
**ASES function raw**						
Wong 2017	117	6	-	>.05	⊖	
**Involved side**						
**SSV raw**						
Cho 2017	46	5	*±-2.27 (-10.12 - 5.58)**	.562	⊗	

Table A16 - Individual results for prognostic factor ‘Symptoms and History’ *(continued)*

Study	n	n other factors	β/OR (95% CI)	p value	RoB	Forest plot
**Previous surgery**						
**SST100 raw**						
Matsen 2019	588	2	-2.9 (-7.1 to 1.3)	-	⊗	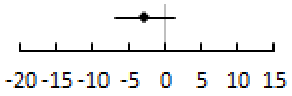
**SST100 %MPI**						
Matsen 2019	588	2	-4.3 (-11.3 to 2.7)	-	⊗	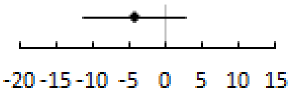
**MEDIUM-LONG TERM**						
**Previous surgery**						
**ASES function raw**						
Shields 2017	272	4	-2.4 (-)	.006	⊗	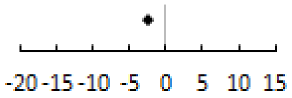
**SSV raw**						
Shields 2017	272	4	-9.2 (-)	<.001	⊗	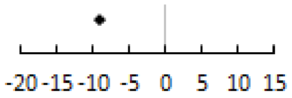
**SSV change**						
Shields 2017	272	4	-11.1 (-)	.003	⊗	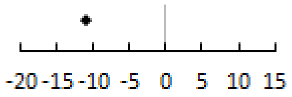
**SST100 raw**						
Matsen 2019	574	2	-4.9 (-9.4 to -.03)	<.05	⊗	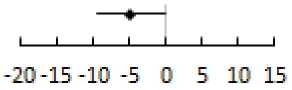
**SST100 %MPI**						
Matsen 2019	574	2	-5.6 (-12 to 0.8)	-	⊗	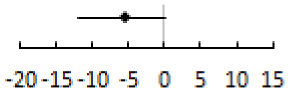
No data for ASES function change (Shields 2017), SST raw / SST change (Okoroha 2019, Patel 2019)						
**Percentage subluxation**						
No data for QuickDASH raw / SANE raw (Dekker 2022)						
SYMPTOMS AND HISTORY ON PAIN						
**MEDIUM-SHORT TERM**						
**Duration of symptoms**						
**VAS pain raw**						
Cho 2017	46	5	-0.002 (-0.007 to 0.004)	.588	⊗	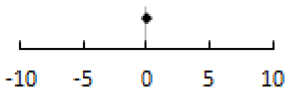
**Dominant side**						
**ASES pain raw**						
Wong 2017	117	6	-	>.05	⊖	
**VAS pain raw**						
Wong 2017	117	6	-	>.05	⊖	
**Involved side**						
**VAS pain raw**						
Cho 2017	46	5	*±0.05 (-1.49 - 1.59)**	.947	⊗	

Study	n	n other factors	β/OR (95% CI)	p value	RoB	Forest plot
**MEDIUM-LONG TERM**						
**Previous surgery**						
**VAS pain raw**						
Shields 2017	272	4	0.09 (-)	.001	⊗	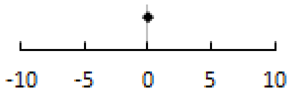
No data available from Patel 2019						
**VAS pain change**						
Shields 2017	272	4	-1 (-)	.011	⊗	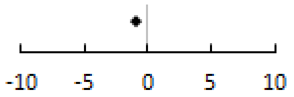
No data available from Patel 2019						
**Dominant side**						
No data for VAS pain raw / VAS pain change (Green 2020)						

* Cho 2017 did not specify whether ‘yes’ or ‘no’ was the reference category, so the direction of effect cannot be interpreted

Table A17 - Individual results for prognostic factor ‘Insurance’

Study	n	n other factors	β/OR (95% CI)	p value	RoB	Forest plot
INSURANCE ON FUNCTIONAL RECOVERY AND PAIN						
**MEDIUM-SHORT TERM**						
**Medicare compared to Medicaid**						
**ASES raw**						
Strotman 2020	91	2	-	<.001	⊗	
**Private insurance compared to Medicaid**						
**ASES raw**						
Strotman 2020	91	2	-	<.001	⊗	
**Private insurance compared to Workers’ Compensation**						
**ASES raw**						
Strotman 2020	91	2	-	.028	⊗	
**Medicare compared to Workers’ Compensation**<br>**Private insurance compared to Medicare**<br>**Workers’ Compensation compared to Medicaid**						
No data available for ASES raw (Strotman 2020)						
**MEDIUM-LONG TERM**						
**Medicare compared to Medicaid**						
**ASES raw**						
Lansdown 2021	324	7	9.8 (-)	.054	⊗	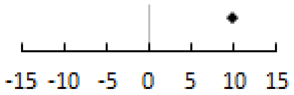
**Private insurance compared to Medicaid**						
**ASES raw**						
Lansdown 2021	324	7	10.5 (-)	.029	⊗	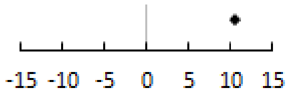
**Private insurance compared to Medicaid/Medicare**						
**ASES top score**						
Forlizzi 2022	162	3	2.7 (1.12 to 6.5)	.03	⊖	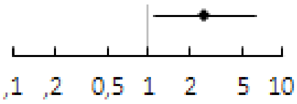
**Workers’ Compensation compared to other insurance**						
**ASES MOI**						
Polce 2021	105	3	0.80 (0.54 to 1.18)	.259	⊗	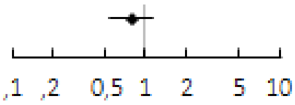

Table A17 - Individual results for prognostic factor ‘Insurance’ *(continued)*

Study	n	n other factors	β/OR (95% CI)	p value	RoB	Forest plot
INSURANCE ON FUNCTIONAL RECOVERY						
**MEDIUM-LONG TERM**						
**Commercial insurance compared to Medicare**						
**SST raw**						
Chawla 2022	33	?	-	>.05	⊗	
**Workers’ Compensation compared to Medicare**						
Chawla 2022	33	?	-	>.05	⊗	
**Workers’ Compensation compared to other insurance**						
**SANE MOI**						
Polce 2021	105	1	0.71 (0.48 to 1.04)	.085	⊗	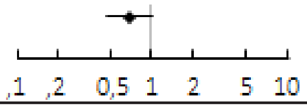
INSURANCE ON PAIN						
**MEDIUM-SHORT TERM**						
**Medicaid compared to Medicare**						
**VAS pain raw**						
Strotman 2020	91	1	-	<.001	⊗	
**Medicaid compared to private insurance**						
**VAS pain raw**						
Strotman 2020	91	1	-	<.001	⊗	
**Private insurance compared to Medicare<br>Workers’ Compensation compared to Medicare<br>Medicaid compared to Workers’ Compensation<br>Private insurance compare to Workers’ Compensation**						
No data for VAS pain raw (Strotman 2020)						

Table A18 – Details on which domains evidence was rated down

			Downgraded on					Upgraded on			
**Time point**	**Domain**	**Prognostic factor**	**RoB**	**Inc**	**Ind**	**Imp**	**PB**	**LE**	**DR**	**PC**	**Certainty**
Medium-short	Functional recovery & pain	Higher age					x				Moderate
	Functional recovery	Male Gender					x				Moderate
		Higher age		x		x	x				Very low
	Pain	Male gender					x				Moderate
		Higher age				x	x				Low
Medium-long	Functional recovery & pain	Walch A2 vs A1									High
		Depression					x				Moderate
		Opioid use				x					Moderate
		Preoperative ASES score		x			x				Low
		Dominant side		x			x				Low
		Previous surgery		x			x				Low
		Male gender		x		x	x				Very low
		Higher age		x		x	x				Very low
	Functional recovery	Male gender					x				Moderate
		Higher age					x				Moderate
		Preoperative SST score				x	x				Low
		Previous surgery				x	x				Low
Long	Functional recovery & pain	Male gender		x		x					Low

RoB = Risk of Bias, Inc = Incompleteness, Ind = Indirectness, Imp = Imprecision, PB = Publication Bias, LE = Large Effect, DR = Dose Response relationship, PC = Plausible Confounding

References

1. Werner BC, Chang B, Nguyen JT, Dines DM, Gulotta LV. What Change in American Shoulder and Elbow Surgeons Score Represents a Clinically Important Change After Shoulder Arthroplasty? Clin Orthop Relat Res. 2016;474(12):2672-81.
2. Michener LA, McClure PW, Sennett BJ. American Shoulder and Elbow Surgeons Standardized Shoulder Assessment Form, patient self-report section: reliability, validity, and responsiveness. J Shoulder Elbow Surg. 2002;11(6):587-94.
3. Franchignoni F, Vercelli S, Giordano A, Sartorio F, Bravini E, Ferriero G. Minimal clinically important difference of the disabilities of the arm, shoulder and hand outcome measure (DASH) and its shortened version (QuickDASH). J Orthop Sports Phys Ther. 2014;44(1):30-9.
4. Liu P, Afzal I, Asopa V, Clement ND, Patel V. Changes and thresholds in the Oxford Shoulder Score following shoulder arthroplasty: Minimal clinically important difference, minimal important and detectable changes, and patient-acceptable symptom state. Shoulder & Elbow. 2023:17585732231176423.
5. Warren JR, Pietroski AD, Franovic S, Ziedas A, Yedulla N, Makhni EC, et al., editors. Characterizing MCID and assessing the role of preoperative PROMIS scores in predicting outcomes for reverse total shoulder arthroplasty at 2-year follow-up. Seminars in Arthroplasty: JSES; 2022: Elsevier.
6. Leggin BG, Michener LA, Shaffer MA, Brenneman SK, Iannotti JP, Williams GR, Jr. The Penn shoulder score: reliability and validity. J Orthop Sports Phys Ther. 2006;36(3):138-51.
7. van Kampen DA, Willems WJ, van Beers LW, Castelein RM, Scholtes VA, Terwee CB. Determination and comparison of the smallest detectable change (SDC) and the minimal important change (MIC) of four-shoulder patient-reported outcome measures (PROMs). J Orthop Surg Res. 2013;8:40.
8. Thigpen CA, Shanley E, Momaya AM, Kissenberth MJ, Tolan SJ, Tokish JM, et al. Validity and Responsiveness of the Single Alpha-numeric Evaluation for Shoulder Patients. Am J Sports Med. 2018;46(14):3480-5.
9. Simovitch R, Flurin PH, Wright T, Zuckerman JD, Roche CP. Quantifying success after total shoulder arthroplasty: the minimal clinically important difference. J Shoulder Elbow Surg. 2018;27(2):298-305.
10. Roy JS, Macdermid JC, Faber KJ, Drosdowech DS, Athwal GS. The simple shoulder test is responsive in assessing change following shoulder arthroplasty. J Orthop Sports Phys Ther. 2010;40(7):413-21.
11. Berglund DD, Law TY, Rosas S, Kurowicki J, Giveans MR, Mijic D, et al. The procedure value index: a new method for quantifying value in shoulder arthroplasty. J Shoulder Elbow Surg. 2019;28(2):335-40.
12. Hallberg K, Salomonsson B. Validity, reliability, and responsiveness of the Swedish version of Western Ontario Osteoarthritis of the Shoulder index. BMC Musculoskelet Disord. 2022;23(1):351.
