# Supplementary material for: Patient-related prognostic factors for function and pain after shoulder arthroplasty: a systematic review
Source: Syst Rev. 2024 Nov 22;13:286. doi: 10.1186/s13643-024-02694-y (PMC11583791; doi:10.1186/s13643-024-02694-y)

Appendix 2 – Supplementary figures

Figure A1 – Meta-analysis for 'Age' on 'ASES raw scores', medium-long term (sensitivity analysis)

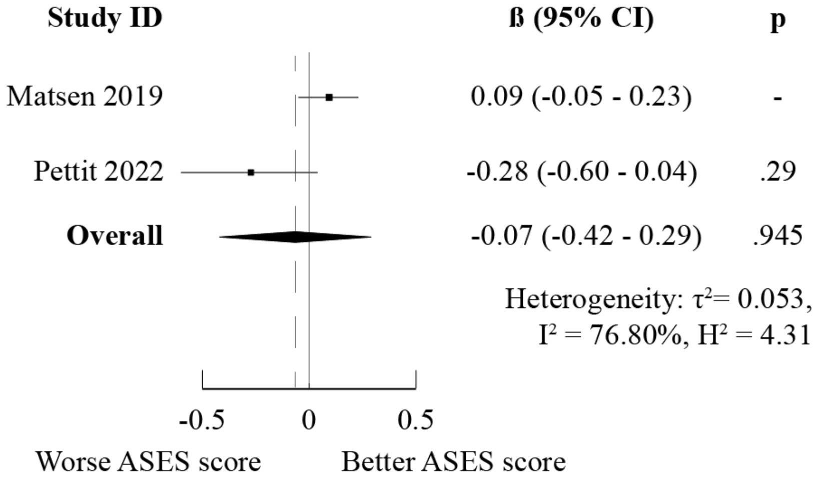

Figure A2 – Meta-analysis for 'Previous surgery' on 'Poor ASES score', medium-long term (sensitivity analysis)

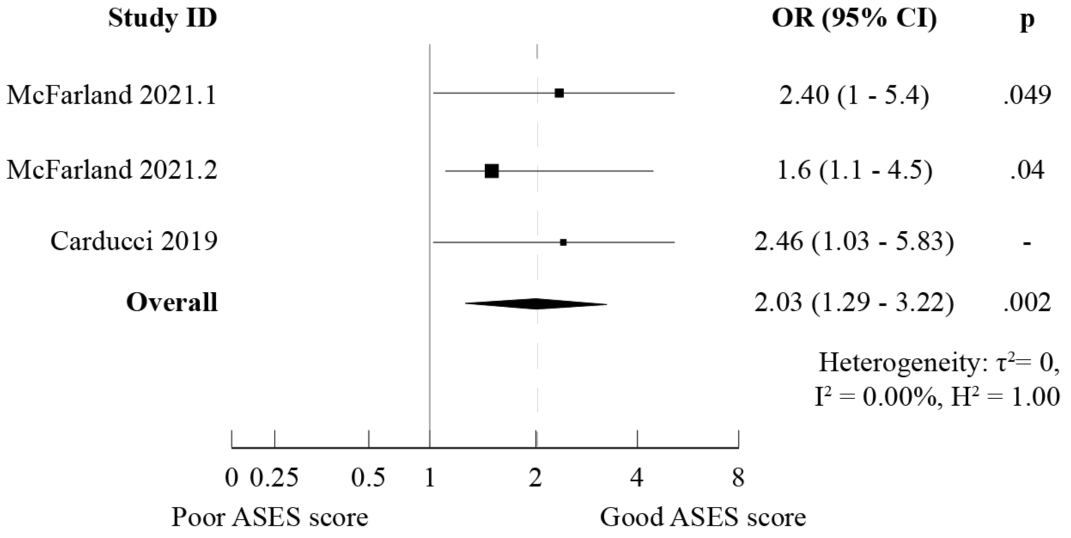

Figure A3 – Summary of Findings figure for Functional Recovery, short term

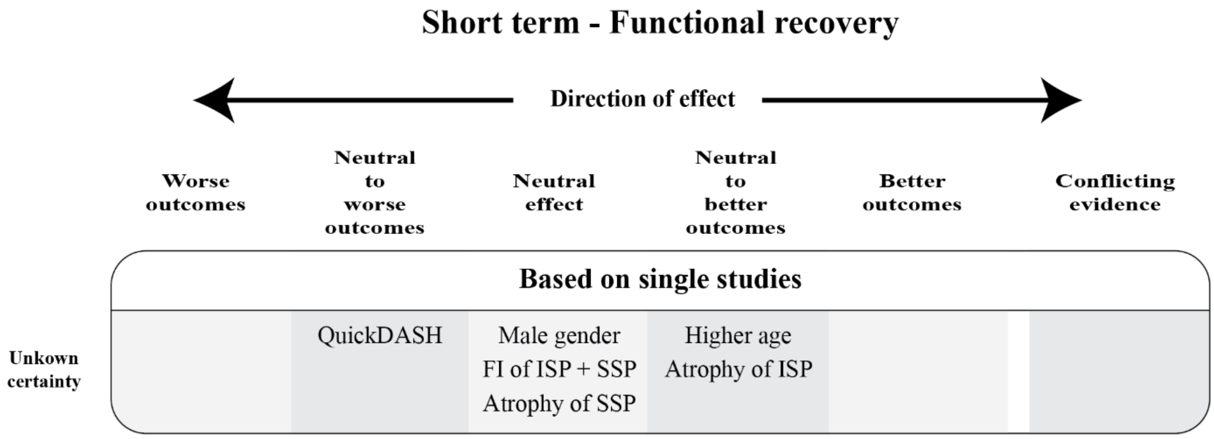

Figure A4 – Summary of Findings figure for Functional Recovery & Pain, medium-long term

Medium-long term - Functional recovery & pain

← Direction of effect →

Based on ≥ 2 studies

	Worse outcomes	Neutral to worse outcomes	Neutral effect	Neutral to better outcomes	Better outcomes	Conflicting evidence
**High certainty**			Walch A2 vs A1			
**Moderate certainty**		Depression<br>Opioid use				
**Low certainty**		ASES<br>Dominant side<br>Previous surgery				
**Very low certainty**		Male gender	Higher age			

Based on single studies

	Worse outcomes	Neutral to worse outcomes	Neutral effect	Neutral to better outcomes	Better outcomes	Conflicting evidence
**Unkown certainty**	No. of patient-reported allergies<br>Back pain<br>Living alone	Higher ASA<br>RCA/RCT vs DJD<br>Diabetes<br>Functional somatic disorder<br>Anxiety<br>WC vs other insurance	OA vs CTA<br>Confidence in achieving outcomes<br>Walch B1 vs A1 / B1 vs A2 / B3 vs A2 / C vs A1<br>Concentric wear vs eccentric wear<br>Glenoid retro-version > 15°<br>Glenoid version per 10°<br>Hamada grade	RCA vs other diagnosis	OA vs other diagnosis<br>Private insurance vs Medicaid / Medicare	Glenoid anteversion

Unclear effect

Higher BMI, comorbdities (unspecified), CCI, smoking, atrophy of SSP, glenoid inclination, expectation ‘relieving nighttime pain’, no. of very important expectations, college education, home zip code median income, marital status, level of activity at work, percent subluxation, Medicare/private insurance vs Medicaid

Figure A5 – Summary of Findings figure for Functional Recovery, medium-long term

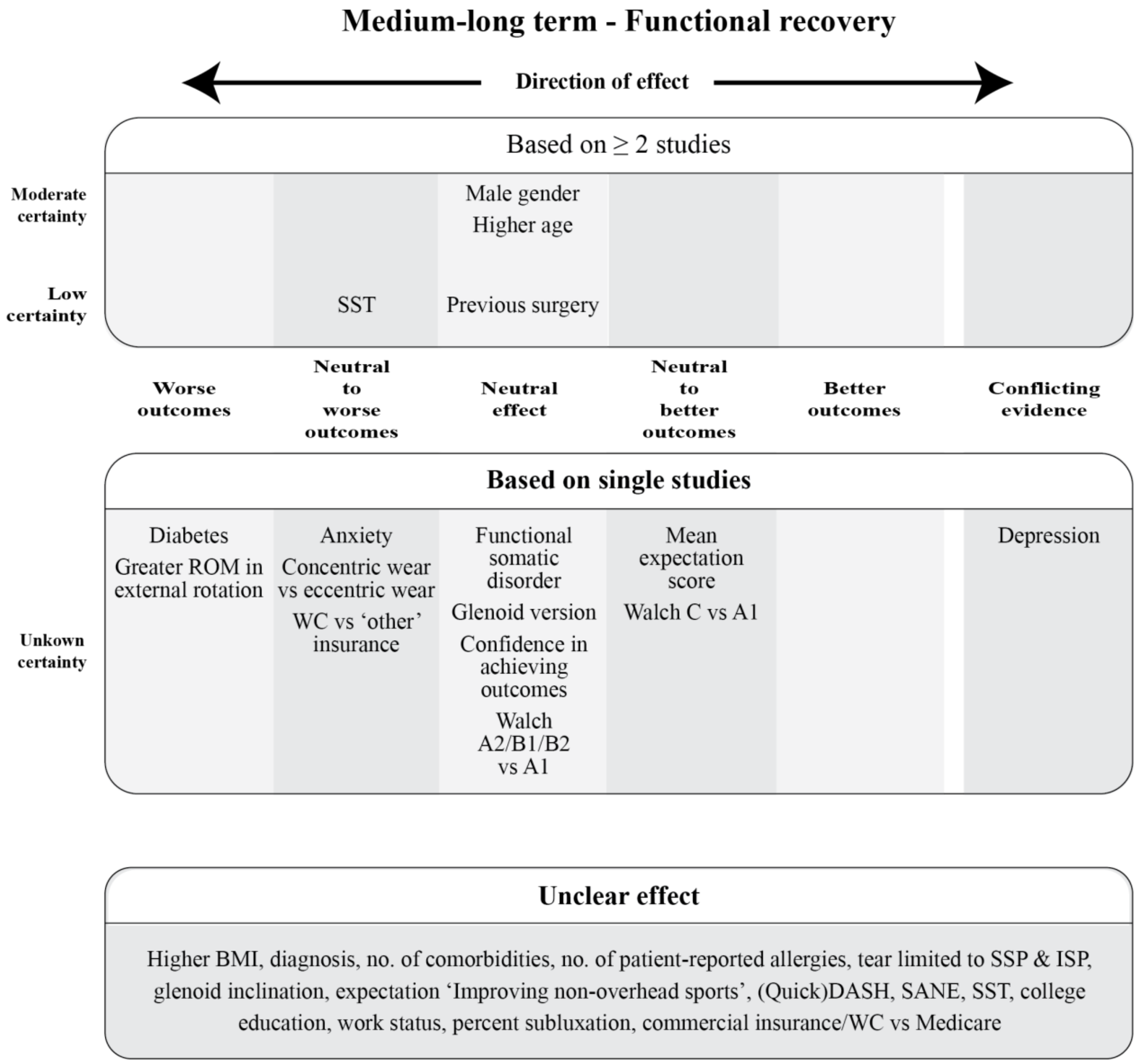

Figure A6 – Summary of Findings figure for Pain, medium-long term

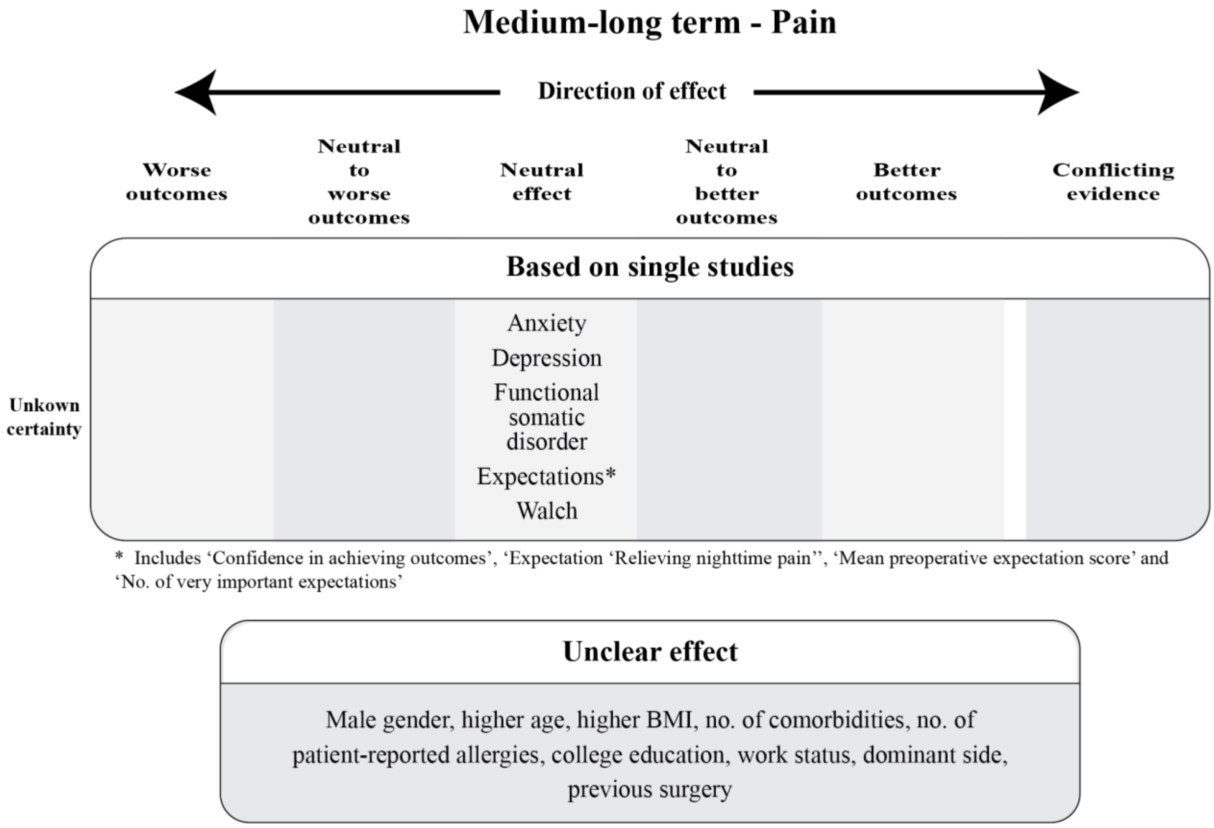

Figure A7 – Summary of Findings figure for Functional Recovery & Pain, long term

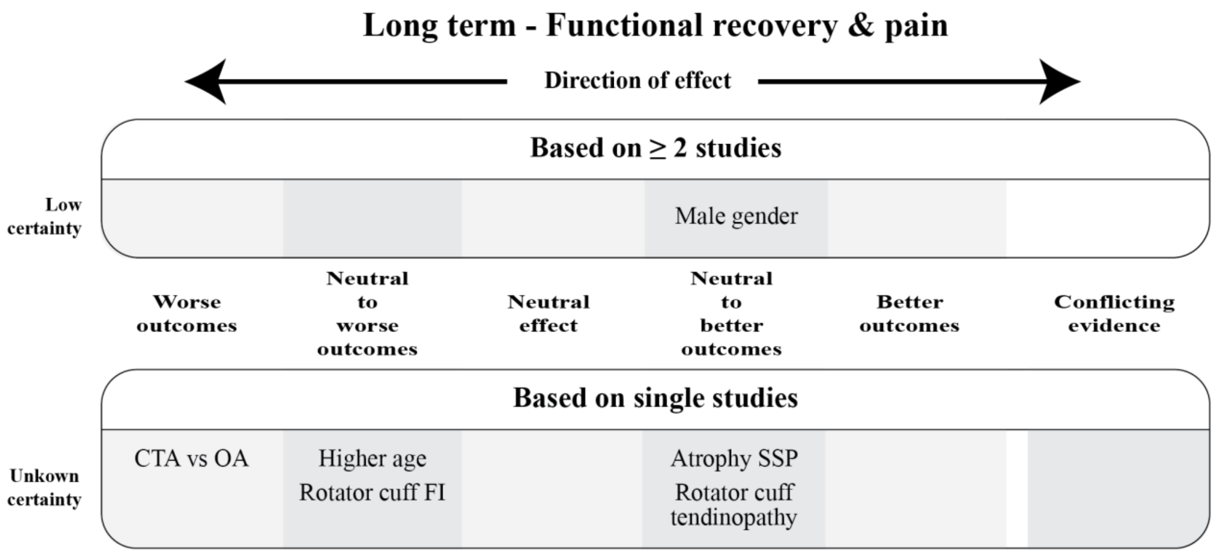

Figure A8 – Summary of Findings figure for Functional Recovery, long term

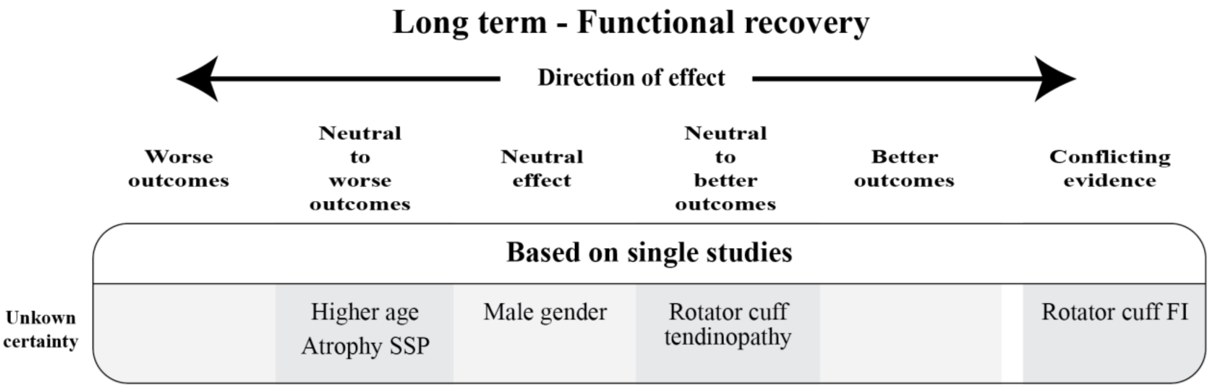

Supplement: Supplementary file 2 — Additional file 2: Appendix 2: Supplementary figures. [file 13643_2024_2694_MOESM2_ESM.pdf]
